# Synergistic Antibacterial Effects of Amoxicillin and Gold Nanoparticles: A Therapeutic Option to Combat Antibiotic Resistance

**DOI:** 10.3390/antibiotics12081275

**Published:** 2023-08-02

**Authors:** Rosa M. Giráldez-Pérez, Elia M. Grueso, Alfonso Carbonero, Juan Álvarez Márquez, Mirian Gordillo, Edyta Kuliszewska, Rafael Prado-Gotor

**Affiliations:** 1 Department of Cell Biology, Physiology and Immunology, Faculty of Sciences, University of Cordoba, 14014 Cordoba, Spain; b82almaj@uco.es; 2 Department of Physical Chemistry, Faculty of Chemistry, University of Seville, 41012 Seville, Spain; pradogotor@us.es; 3 Department of Animal Health, Veterinary Faculty, University of Cordoba, 14014 Cordoba, Spain; sa1camaa@uco.es (A.C.); v82gomam@uco.es (M.G.); 4 Chemtra Company, 47-300 Krapkowize, Poland

**Keywords:** gold nanoparticles, antibiotic resistance, aureus nanosystem, gemini surfactant, amoxicillin

## Abstract

Compacted Au@16-mph-16/DNA-AMOX (NSi) nanosystems were prepared from amoxicillin (AMOX) and precursor Au@16-mph-16 gold nanoparticles (Ni) using a Deoxyribonucleic acid (DNA) biopolymer as a glue. The synthesized nanocarrier was tested on different bacterial strains of *Escherichia coli*, *Staphylococcus aureus,* and *Streptococcus pneumoniae* to evaluate its effectiveness as an antibiotic as well as its internalization. Synthesis of the nanosystems required previous structural and thermodynamic studies using circular dichroism (CD) and UV-visible techniques to guarantee optimal complex formation and maximal DNA compaction, characteristics which facilitate the correct uptake of the nanocarrier. Two nanocomplexes with different compositions and structures, denoted NS_1_ and NS_2_, were prepared, the first involving external Au@16-mph-16 binding and the second partial intercalation. The Ni and NSi nanosystems obtained were characterized via transmission electron microscopy (TEM), zeta potential, and dynamic light scattering (DLS) techniques to measure their charge, aggregation state and hydrodynamic size, and to verify their presence inside the bacteria. From these studies, it was concluded that the zeta potential values for gold nanoparticles, NS_1_, and NS_2_ nanosystems were 67.8, −36.7, and −45.1 mV. Moreover, the particle size distribution of the Au@16-mph-16 gold nanoparticles and NS_2_ nanoformulation was found to be 2.6 nm and 69.0 nm, respectively. However, for NS_1_ nanoformulation, a bimodal size distribution of 44 nm (95.5%) and 205 nm (4.5%) was found. Minimal inhibitory concentration (MIC) values were determined for the bacteria studied using a microdilution plates assay. The effect on *Escherichia coli* bacteria was notable, with MIC values of 17 µM for both the NS_1_ and NS_2_ nanosystems. The *Staphylococcus aureus* chart shows a greater inhibition effect of NS_2_ and NP_2_ in non-diluted wells, and clearly reveals a great effect on *Streptococcus pneumoniae*, reaching MIC values of 0.53 µM in more diluted wells. These results are in good agreement with TEM internalization studies of bacteria that reveal significant internalization and damage in *Streptococcus pneumoniae*. In all the treatments carried out, the antibiotic capacity of gold nanosystems as enhancers of amoxicillin was demonstrated, causing both the precursors and the nanosystems to act very quickly, and thus favoring microbial death with a small amount of antibiotic. Therefore, these gold nanosystems may constitute an effective therapy to combat resistance to antibiotics, in addition to avoiding the secondary effects derived from the administration of high doses of antibiotics.

## 1. Introduction

Antimicrobial resistance (AMR) is currently one of the biggest threats to global health and food security, according to the World Health Organization (WHO) [1]. A study from data reported by 87 countries in 2020 revealed high AMR levels, which cause potentially fatal sepsis, as well as growing resistance to treatment in various bacteria that cause common infections among the population [2]. Therefore, there is an urgent need to find new, more effective antibiotics to counteract these resistances. However, clinical development of new antimicrobials is very limited. In 2019, thirty-two antibiotics were in the development phase, and only six were classified as innovators [3]. This problem affects countries at all levels of development.

Due to the complexity and breadth of the AMR problem, a unified multisectoral view is required, using the “one health” approach, to establish communication ties and collaborate in the project and implementation of programs, policies, legislation, and research to achieve better public health outcomes [4].

Understanding of the different mechanisms of antimicrobial resistance is very important for th creation of new alternatives to counteract it. Some examples of these resistance mechanisms are avoiding the accumulation of antibiotics, the modification of the target molecules, and the enzymatic inactivation of antibiotics. The first type of mechanism can reduce the absorption of these molecules by modifying the outer bacterial membrane (losing or modifying the porins to prevent antibiotic access), increasing their discharge through the pumps, or both. Hydrophobic antibiotics, such as quinolones and macrolides, pass through the lipid bilayer, whereas hydrophilic antibiotics, such as beta-lactams, pass through porins [5,6]. This may be an innate characteristic of an organism or may be produced by a mutation or acquisition of exogenous resistance genes [7].

One antibiotic that covers a broad spectrum of antimicrobial activity is amoxicillin (AMOX), whose use dates from the 1970s. This semisynthetic penicillin is the most widely used in the world, either alone or combined with clavulanic acid [8]. According to the American Society of Health-System Pharmacists, AMOX prevents bacterial growth. It is used in the treatment of bacterial infections such as pneumonia and bronchitis, and ear, nose, throat, urinary tract, and skin infections.

AMOX binds to penicillin-binding protein (PBP) 1A, an enzyme that is essential for the synthesis of the bacterial cell wall. The β-lactam ring of amoxicillin modifies the carbon terminus of the PBP 1A transpeptidase. It forms an irreversible union that prevents the enzyme from carrying out its function of synthesizing peptidoglycan, which makes up the bacterial wall. This will ultimately result in an alteration of the membrane and an increase in its permeability, leading to cell lysis and death [8]. AMOX has a hydroxyl group that makes it more soluble in lipids, and therefore gives it greater bioavailability, duration of action, and bactericidal activity. AMOX is normally administered orally, and it is usually rapidly absorbed, presenting greater bioavailability. Adverse effects must also be considered. In this sense, AMOX presents some toxicity, sometimes causing lesions in the bile ducts [9], as well as hypersensitivity reactions [10].

The resistant bacteria used in this work are *Escherichia coli (E. coli)*, *Streptococcus pneumoniae* (*S. pneumoniae*) and *Staphylococcus aureus (S. aureus*). *E. coli* is a Gram-negative bacterium that is classified as a member of the Enterobacteriaceae family. *E. coli* includes not only commensal but also pathogenic strains that cause a variety of human diseases, resulting in millions of deaths each year [11] due to virulence factors and pathogenicity mechanisms that cause gastrointestinal diseases like diarrhea [11]; other strains can cause haemolytic uraemic syndrome and hemorrhagic colitis [11], leading to acute renal failure, which can cause death. *E. coli* also has a large environmental impact on water quality and public health [12]. *S. aureus*, a Gram-positive microorganism, can survive in very adverse conditions, easily colonizing the skin and therefore penetrating tissues. The most frequent pathologies of *S. aureus* are infections of the skin and soft tissues, otitis, osteomyelitis, arthritis, pneumonia, and sepsis [13], generating the majority of nosocomial diseases. *S. pneumoniae* is a Gram-positive bacterial pathogen that may asymptomatically colonize the upper respiratory tract and can cause infections including conjunctivitis, otitis media, lower respiratory tract infections, bacteremia, and meningitis [14,15]. The mechanisms of antibiotic resistance of *S. pneumoniae* include the evolution of resistance patterns and mechanisms for beta-lactam antibiotics, among others [16,17].

Knowledge of the different mechanisms of resistance to antimicrobials is essential to be able to propose new strategies that solve the problem of resistance to antibiotics. Among the different mechanisms studied are those based on the production of enzymes that are inactivating [18]; there are also those based on modifications of the therapeutic target, and others that act by decreasing the intracellular concentration of the antibiotic [18]. The mechanisms of intracellular antibiotic reduction may be due to a mechanism based on efflux pumps that expel the antibiotic [19], or a mechanism that modifies the external bacterial membrane, so that the bacteria lose or modify porins, thereby preventing the entry of the antibiotic. An example of these are beta-lactams, hydrophilic antibiotics that cross porins [20], or the use of vehicles made up of gold nanoparticles (AuNPs) that can interact with the exterior of the microorganism, destabilizing its exterior [21].

Nanoparticles have numerous biological, biomedical, pharmaceutical, and nutritional applications [22]. Materials at the nanoscale (1–100 nm) display unique physicochemical properties [23], and their small size allows them to penetrate bacterial membranes (>1 µm). Some studies also show that metal nanoparticles have antimicrobial activity in themselves, especially metal nanoparticles, with the advantage that microorganisms are not able to create resistance to such materials. Nanosystems are based on the idea of the Trojan horse, and have been highly successful in the pharmaceutical industry because they exhibit stable drug loading, expanded pharmacokinetics, reduced off-target side effects, and improved efficiency of drug delivery through their ability to penetrate blood–brain barriers or plasma membranes [24].

In recent years, there has been increasing interest in the development of optimal metal-base nanoparticles (MBNPs) and their usage synergy with antibiotics for combating antibiotic-resistant bacteria [25]. In this context, MBNPs–antibiotic/biopolymer biomaterials have been demonstrated to be crucial due for the potential synergistic functionalities of these kind of complexes, which are different from those presented in their individual counterparts. Forming part of these nanocomplexes, distinct oxides like TiO [26,27], ZnO [28,29,30], and the nanometallic Ag [30,31,32,33], among others, are specifically found in the bibliography. Metallic nanomaterials affect the bacterial cell membrane, and they can release antibacterial metal ions, generate reactive oxygen species, inhibit enzymatic activity and DNA synthesis, and interrupt energy transmission [34]. However, the principal mechanism of action of MBNPs includes the production of oxidative stress, interaction with cell membrane, or the release of ions [25]. This occurs n such a way that these new nanomaterials not only improve the sustained release of the antibiotic, but also enhance the antibacterial effect of the drug in comparison with the conventional usage of the free antibiotic. For instance, Ag-based nanosystems can act through multiple mechanisms, depending on the specific nanoformulation. Thus, they can act by generating oxidative stress [31], interacting with the cellular membrane without damaging the outer membrane [32], thereby contributing to the release of ions and preventing DNA replication [33], or acting as enzyme inhibitors [30], such is the diversity of the mechanisms studied to date. In contrast, the mechanism of action of Au-based nanosystems has been less explored, and the majority of the studies in the bibliography indicate that the mechanism of the cations of these kind of metal nanoparticles is fundamentally based on oxidative stress oxidation [35]. However, AuNPs offer certain advantages over other metallic nanoparticles. As is known, AuNPs improve nanosystem biocompatibility and protect against enzymatic degradation exerted on biomolecules like DNA or ribonucleic acid (RNA) [36]. Thus, the combined use of AuNPs and the DNA biopolymer contributes to overcoming problems related to biocompatibility. Moreover, the small size of the synthesized nanoformulations makes them capable of diffusing effectively through the bacterial cell wall, thus improving internalization and interaction with the bacteria, as has been demonstrated in previous works [37,38].

Therefore, the main objective of this work is to obtain stable and biocompatible Au@NPs/DNA-AMOX nanocomplexes for evaluating their synergistic antibacterial effects caused by both the precursors and the configured Au@16-mph-16/DNA-AMOX nanosystems. In this regard, 16-mph-16 is a biocompatible and biodegradable gemini surfactant with a benzene spacer that shows great antibacterial properties against distinct bacteria and microscopic fungi. For instance, the MIC values of the analogous 16-Ph-16 against *E. coli* and *S. aureus* bacteria were found to be 0.3906 and 1.5625 mM, respectively [39]. Moreover, this type of surfactant has proven to be very efficient in DNA compaction, interacting strongly with DNA via partial intercalation [40]. Thus, the combination of both the antimicrobial properties of 16-mph-16-covered AuNPs and a DNA-AMOX complex in a unique nanosystem results in a nanoformulation that acts quickly, favoring microbial death with a small amount of antibiotic, thereby combating resistance to antibiotics in addition to avoiding the secondary side effects derived from the administration of high doses of antibiotics. 

## 2. Materials and Methods

### 2.1. Materials

All chemical products are of high purity and have been used without further purification. Deoxyribonucleic acid (DNA) from calf thymus, amoxicillin (AMOX), hydrogen (III) tetrachloroaurate trihydrate (HAuCl_4_), sodium cacodylate, and 3-aminopropyltriethoxylan (APTES) were purchased from Sigma-Aldrich–Merck KGaA (Darmstadt, DEU); sodium borohydrate (NaBH4) was purchased from Panreac Química S.L.U. (Barcelona, ES). The DNA was used without further purification, controlling the absorbance ratio of the DNA stock solutions at 260 nm and 280 nm, resulting in values between 1.8 and 1.9 (A_260_/A_280_ = 1.87), indicating that there is no protein contamination [41]. An ethidium bromide agarose gel electrophoresis test indicated that the mean number of base pairs per DNA molecule was greater than 10,000 b.p. To establish biopolymer concentrations in base pairs, ds-DNA concentrations were determined spectrophotometrically at 260 nm from 13,200 M^−1^cm^−1^ molar absorptivity of DNA [42]. To control the size of the nanosystems, the DNA was cut with a Bioruptor, at 700 b.p. The total concentrations of the DNA polynucleotide, AMOX, 16-mph-16 gemini surfactant, 16-mph-16 nanoparticles, Au@16-mph-16, and the compacted nanosystem, Au@16-mph-16/DNA-AMOX in a working solution will now be referred to as C_DNA_, C_AMOX_, C_16-mph-16_, C_Au@16-mph-16_, and C_Au@16-mph-16/DNA-AMOX_, respectively. All solutions were prepared with deionized and autoclaved water (conductivity less than 10^−6^ S·m^−1^) at a fixed ionic strength of 1.63 mM.

#### 2.1.1. Bacterial Lines and Culture Conditions

Commercial reference strains were used to test the effect of AuNPs and nanosystems. Specifically, *Staphylococcus aureus* ATCC^®^ 29213 (Thermo Scientific, Lenexa, KS, USA), ATCC^®^ 25922 (Thermo Scientific, Lenexa, KS, USA) and *Streptococcus pneumoniae* ATCC^®^ 49619 (Thermo Scientific, Lenexa, KS, USA) were used. The three bacteria were grown under aerobic conditions at 37 °C. The bacterial growth in Mueller Hinton blood agar is shown in Figure S1. These three species are currently responsible for the great majority of human deaths caused by bacterial agents (GBD, 2029).

#### 2.1.2. Synthesis of 16-mph-16 Surfactant

**Scheme 1:**
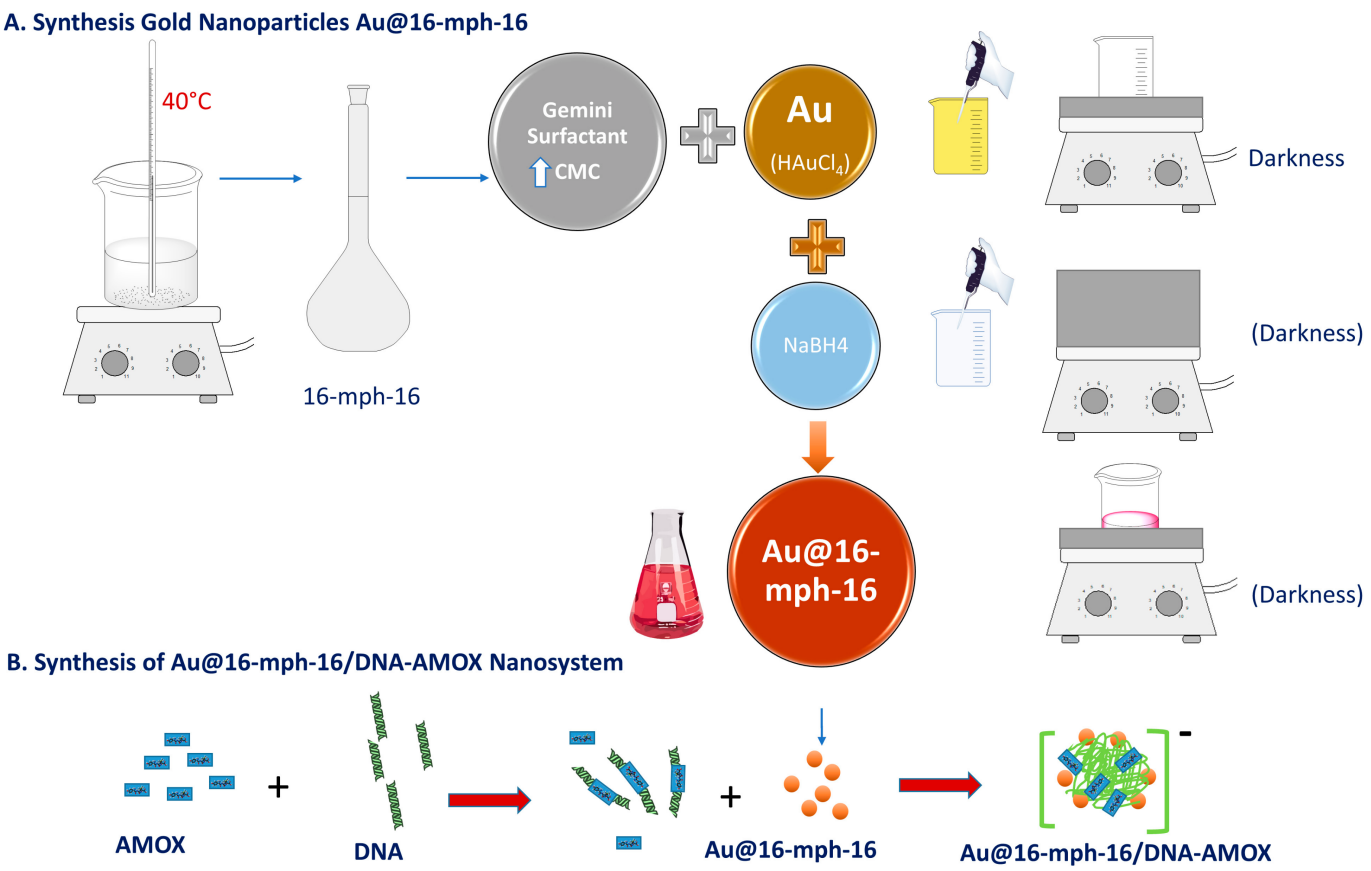
(**A**) Schematic representation of the synthesis of Au@16-mph-16 nanoparticles. (**B**) Schematic representation of the synthesis of Au@16-mph-16/DNA-AMOX nanosystem (for more details, see Graphic Abstract).

The synthesis and characterization of the 16-mph-16 (for more details, see Figure S2 and the NMR characterization data in the Supporting Information section) was carried following the procedure described in a previous work [40]. Its measured CMC was (7.0 ± 0.3) × 10^−6^ M, via the surface tension technique. To prepare the 16-mph-16 surfactant solution necessary for the synthesis of the 16-mph-16-stabilized gold nanoparticles, Au@16-mph-16, a surfactant concentration five times higher than CMC in water (0.035 × 10^−3^ M), was employed. A sonicator was used to facilitate the dissolution of 16-mph-16 in the solvent solution for 2 min. Once this was complete, Milli-Q water was heated to 40 °C; then, maintaining this temperature, the solution was continuously stirred until crystal clear, to ensure that the surfactant had completely dissolved. The surfactant must be at room temperature for use (Scheme 1A).

#### 2.1.3. Synthesis of Au@16-mph-16 Nanoparticles

Synthesis of gold nanoparticles coated with the biodegradable hydrophobic gemini surfactant 16-mph-16 was taken as a starting point; therefore, the cationic Au@16-mph-16 is a precursor of the nanosystem. To prepare 16-mph-16-functionalized AuNPs, 760 μL of 23 mM HAuCl_4_ aqueous solution at 99.9% purity was added to 60 mL of 10^−4^ M 16-mph-16 surfactant, and the mixture was shaken vigorously for 5 min in darkness, yielding a yellow solution. Subsequently, 200 μL of a freshly prepared aqueous solution of 0.4 M NaBH_4_ at 96% purity was added dropwise to the previously prepared mixture, and stirred moderately for 10 min in darkness, then acquiring a crystalline reddish color. It was found that the optimal condition for synthesizing AuNPs was 24 h of rest time at 5.0 °C. As a result, an aqueous solution of Au@16-mph-16 nanoparticles was obtained at a concentration of 1.70 × 10^−7^ M. In this study, for bacterial experiments, we used two Au@16-mph-16 (Ni) formulations for bacterial experiments prepared at different C_16-mph-16_ concentrations of 3.4 nM and 32.6 nM, which were named N_1_ and N_2_, respectively (Scheme 1A).

#### 2.1.4. Synthesis of Au@16-mph-16/DNA-AMOX Nanosystem

The appropriate Au@16-mph-16 concentration used to prepare the nanosystems was previously determined using CD technique to guarantee the maximum compaction of the DNA/AMOX complex bound to the Au@16-mph-16 precursor, where small DNA chains (700 b.p.) were used. For this, the values found in the CD studies were used. In addition, DNA-AMOX complexes were prepared by mixing for 2 min at room temperature, working under saturation conditions to transport the maximum amount of drug per nanocomplex (X = C_AMOX_/C_DNA_ = 0.5) and using the highly stable DNA-AMOX complex as a vehicle [43]. The prepared DNA-AMOX complex was gently shaken with Au@16-mph-16 nanoparticles and incubated at 25 °C for 5 min. As a result, the position of the surface plasmon resonance (SPR) absorbance peak moved from 517 nm to 518 nm. This change was accompanied by an increase in the absorbance intensity of the nanoparticle after 24 h of stabilization time and cold conditioning, which is indicative of the formation of the nanosystems. In NS_1_ and NS_2_, a C_DNA_ = C_AMOX_ = 68 μM was used, with C_N1_ being 3.4 nM and C_N2_ being 32.6 nM (Scheme 1B and Graphical Abstract).

### 2.2. Methods

#### 2.2.1. DNA Fragmentation

DNA was sheared for seven cycles of 20 s ON/30 s OFF with the Bioruptor^®^ Pico (Diagenode Co., Liege, Belgium) using 100 µL (0.5 mL Bioruptor^®^ tubes (Diagenode Co., Liege, Belgium)). The control and set-up to obtain DNA of adequate size was carried out by means of agarose gel electrophoresis, with a complete electrophoresis system (PowerPac™ Basic Power Supply #1645050. BIO-RAD, Hercules, CA, USA). The gel makes it possible to separate charged molecules based on their size and shape. To do this, a molecular weight marker (1350–50 p.b.) was used with DNA fragments of known size, so that the approximate size of the DNA under study could be calculated.

#### 2.2.2. UV/Vis Spectroscopy

To measure the absorbance spectra, a Zuzi 4255/50 co. double beam optical system was used (Zuzi, STL DASELAB, S.L, Valencia, ES). Data were collected every 2 nm using a standard quartz cell with a path length of 10 mm. The wavelength precision and spectral bandwidth were ± 0.3 nm and 0.5 nm, respectively. To study the stability of the Au@16-mph-16 and Au@16-mph-16/DNA-AMOX nanosystems, changes in the UV-vis spectra from 200 to 800 nm were followed over time and checked for at least 1 month. To study the formation of Au@16-mph-16/DNA-AMOX complexes, we fixed both DNA and AMOX concentrations at C_DNA_ = C_AMOX_ = 68 μM, and followed the changes in the maxima of the Au@16-mph-16 SPR band at 518 nm with varying gold nanoparticle concentrations from 0.28 to 40.9 nM. Drug release studies using AMOX from the NS_1_ and NS_2_ nanocomplexes were carried out by monitoring the time-dependent release profiles in Mueller Hinton medium at 37 °C to mimic the bacterial environment. To achieve this, 1.5 mL of the NS_1_ or NS_2_ nanosystem was mixed and dispersed in 1.5 mL of Mueller Hinton, and the kinetics traces were measured and recorded at 230 nm and 520 nm wavelengths using the spectrophotometric technique. The quantity of AMOX released from the nanocomplexes was assessed from the kinetic curve after correction from the DNA contribution at 230 nm and using the appropriate AMOX calibration curve measured in the same solvent condition (water: Mueller Hinton medium (1:1, *v:v*)). The percentage of unreleased AMOX (%UR-AMOX) was calculated as follows: %UR-AMOX = ((CAMOX encapsulated − CAMOX released)/CAMOX en-capsulated) × 100. Each experiment was repeated at least five times, and the maximum spread of time constants was found to be within 10%. To understand the kinetic behavior, drug release profiles were fitted according to distinct kinetic models, meaning that the better curve fit was obtained using the bi-exponential first order equation.

#### 2.2.3. Circular Dichroism Spectroscopy (CD)

A study of the interactions as well as the conformational changes induced by Au@16-mph-16 nanoparticles in DNA/AMOX complexes was carried out with electronic CD spectra using a BioLogic Mos-450 spectropolarimeter (Barcelona, ES). For this purpose, fixed concentrations of C_DNA_ = 68 μM and C_AMOX_ = 68 μM and varying concentrations of Au@16-mph-16 (from 0.37 nM to 23.3 nM) were used. The samples were deposited in a standard quartz cell with a path length of 1 cm. The spectra were expressed in terms of molar ellipticity [θ]. Measurements were taken from 210 nm to 320 nm, working in the intrinsic CD region of DNA. For each spectrum, six to ten scans were averaged at a constant temperature of 298.0 K, with a 10 min equilibration before each scan.

#### 2.2.4. Zeta Potential Measurements and Dynamic Light Scattering (DLS)

To control the size distribution of the different synthesized Ni and NSi nanoformulations, a characterization was performed using dynamic light scattering (DLS) with a Zetasizer Model ZS-90 (Malvern, Worcestershire, UK). Particle size distribution was measured using laser diffraction. To achieve this, the angular variation of the intensity of scattered light is measured when a laser beam passes through a sample of scattered particles; by analyzing the data of the angular scattering intensity, we can calculate the size of the particles that create the scattering pattern. At least five size measurements were taken for each sample, calculating the relative error for the hydrodynamic diameter, which was <5%. Light scattering measures the electrophoretic mobility of particles in dispersion or molecules in solution (Figure S4). This mobility is converted to the zeta potential (ζ). For this, we used a Zetasizer Nano ZS from Malvern Instrument Ltd. (Worcestershire, UK). A laser Doppler velocimeter (LDV) and DTS1060 polycarbonate cuvettes were used. The number of measurements was at least six per sample. To prepare the samples, the concentrations of C_16-mph-16_ were varied, while the concentrations of 6.8 × 10^−5^ M C_DNA_ and 6.8 × 10^−5^ M C_AMOX_ were fixed (Figure S5).

#### 2.2.5. Transmission Electron Microscopy (TEM)

To obtain TEM images of the Au@16-mph-16 gold nanoparticles, a high-resolution electron microscope TEM TALOS^TM^ F200S (FEI Co., Hillsboro, OR, USA) was used. The sample was deposited on a copper grid covered with a carbon film, then dried in a vacuum pump for at least 30 min. The resulting images were analyzed using ImageJ 1.52a free software (1997–2018, https://imagej.net/ij/index.html; accessed on 13 June 2023), and the diameter of 350 nanoparticles was measured.

A Zeiss Libra 120 electron microscope (Zeiss, Jena, DEU) was used to visualize both the precursor and compacted nanosystems in cell samples. The study was carried out with about 450 cells for each treatment used, including free AMOX, Au@16-mph-16 (N_2_) nanoparticles, Au@16-mph-16 compact nanosystems/DNA-AMOX (NS_2_), as well as controls without any reagent and with Mueller Hinton culture medium. For the fixation of the different bacterial samples, a 2.5% glutaraldehyde solution was used. Subsequently, the samples were washed several times with a cacodylate trihydrate solution (0.1 M and pH: 7.4) for 1 h at room temperature and/or 277.0 K overnight. Using an automatic sample processor, they were treated for 33 h and 25 min. Finally, the samples were treated with a 1% osmium tetroxide solution. For contrast and staining of the samples, a uranyl acetate solution with a concentration of 2% was used. Subsequently, the samples were dehydrated and gradually embedded in epoxy resin. They were then maintained at 343.0 K for 7 h for polymerization of the resins. The prepared samples were then ready for cutting. First, semi-thin sections were made with a glass slide in a standard range of 300 nm to determine the best areas for study; for this purpose, the sections were stained with toluidine blue and visualized with an optical microscope. Ultrafine sections (less than or equal to 70 nm) were then made with a diamond-edged blade, and the sections deposited on 300 mesh copper grids. Visualization of the samples was performed with a Zeiss Libra microscope. For more details, see the protocol followed by the research group in previous works [21,40,44,45,46]. The different samples were studied by observation of between 500 to 700 bacteria per experiment.

#### 2.2.6. Energy-Dispersive Spectroscopy (EDS) Measurements

For the study of elementary components, we prepared cells fixed, treated, and cut with the ultramicrotome. Next, a microanalysis of an ultrathin section of the sample was carried out using the electronic scanner of the Zeiss EVO microscope (Zeiss, Jena, DEU). To do this, we used energy-dispersive spectroscopy (EDS), with which we determined the presence of gold in the sample.

To analyze the sample and verify the presence of gold, we used a Zeiss Crossbeam 550 microscope (Zeiss, Jena, DEU), which enables image analysis using a high-resolution field emission scanning electron microscope (FE-SEM) in combination with the processing capability of an ion beam (IFB). This microscope also allows for EDS mapping with a very high spatial resolution by reducing the interaction volume when the sample sections are thin. The STEM microscope performed with an SEM is considered low-voltage STEM, since the highest possible accelerating voltage of the electron beam in SEM is 30 kV, i.e., much lower than most modern TEMs. Images can be used as bright field (BF), annular dark field (ADF), and high angle annular dark field (HAADF). Each mode captures a different set of electron signals and offers a very different contrast. The energy-dispersive X-ray spectroscopy (EDS) was performed in STEM mode. The advantage of this compared to EDS in SEM mode is that it offers much higher resolution and less interference due to the greatly reduced volume of interaction, given the limited thickness of the sample sections. In this case, 150 nm sections of samples embedded in epoxy resin were made and deposited on copper grids. In other cases, Si slides covered with indium tin oxide (ITO)-coated glass covers were used (SE Supplies LLC, Tucson, AZ, USA). The resolution was 0.7 nm at 30 kV (STEM mode), 1.6 nm at 1 kV (SEM mode), 3 nm at 30 kV (FIB), with 12×–2,000,000× (SEM) magnification, 300×–500,000× (FIB), one accelerating voltage: 0.02–30 kV (SEM) −0.5–30 kV (FIB) and a current probe: 10 pA to 283 nA (SEM) −1 pA to 100 nA (FIB).

#### 2.2.7. Gold Nanosystems’ Susceptibility Tests against Reference Strains

After selection of the most stable nanosystems, the comparative antibacterial efficacy was evaluated in vitro on bacterial cultures. Each bacterial system selected was explored in the presence of AMOX (102 μL of amoxicillin 1.73 mM in 2.898 mL of water), nanoparticle N_1_ (23.8 mL of NP in 376.8 mL of molecular water; C_16-mph-16_ concentration of 3.4 nM), nanoparticle N_2_ (233.6 mL of NP in 166.4 mL of molecular water; C_16-mph-16_ concentration of 32.6 nM)), and the NS_1_ and NS_2_ nanosystems (where C_DNA_ = C_AMOX_ = 68 μM was fixed and C_16-mph-16_ concentrations were varied, being 3.4 nM and 32.6 nM for NS_1_ and NS_2_, respectively). In this way, the minimum dose necessary to inhibit bacterial growth was evaluated in each case. To evaluate the effectiveness of the selected nanosystems on bacterial growth to find the most suitable one, their minimum inhibitory concentrations (MICs) were determined using a two-fold micro-dilution standard assay, according to the protocol described by the European Committee on Antimicrobial Susceptibility Testing (EUCAST). The cutoff values used for the interpretation of MIC results were taken from EUCAST [47,48]. For the assays, 96-well U-bottom plates were used, with one row being used to assess the effect of the two nanosystems, another two for each nanoparticle concentration (not attached to AMOX), another row for AMOX, and a final row with only Mueller Hinton broth (the negative control row).

Some 100 µL of all of these was dispensed in each of the first wells of each row of the plate, followed by double dilutions in 50 µL of Mueller Hinton broth that had been previously dispensed in the rest of the columns (2–8). To this end, 50 µL was taken from each well, starting with the first, and passed to the well in the second column, leaving the product diluted by half. From this, 50 µL was passed to the third and so on until the last, from which 50 µL was discarded in order to always end up with a final volume of 50 µL. Finally, 50 µL of a bacterial suspension with an optical density of 0.08–0.1 (approximately 105 cfu) was added to all wells. The plates were sealed with parafilm and incubated for 24 h at a temperature of 37 °C. After this period, a macroscopic control of the plates was performed, and the dilution at which a button of bacteria was observed in the bottom was verified. The plates were then shaken on a shaker, and once all the buttons had disappeared, each well was read spectrophotometrically in an ELISA reader at a wavelength of 540 nm.

This protocol was used three times for each bacterium, such that the results for each well are the average of readings for the three plates. In addition, three “blank” plates were prepared, using the same protocol but without the addition of the bacteria in the last step. These plates were also incubated and analyzed in the same way.

Optical density values at 540 nm for distinct nanosystems were corrected for the contribution of AuNPs themselves in the absence of bacteria, and for the contribution of Mueller Hinton medium at the same wavelength of measurement. The correction was made considering the corresponding dilution used in each experiment.

#### 2.2.8. Evaluation of Nanoparticles and Nanosystems on Agar Plates

At this point, tests were carried out to evaluate the effect of the nanoparticles and nanosystems on the three bacteria used in the study.

Agar plates of Mueller Hinton agar with added sheep blood were used to culture *E. coli*, *S. aureus* and *S. pneumoniae*. After this, and before incubation, the plates were divided into four parts (see Figure S8). To each part, a 100 µL drop of the following solutions was added: concentrated nanoparticle, nanoparticle in a 0.5 dilution, nanoparticle in a 0.25 solution and an amoxicillin disk (25 µg). After a 5 min pause to allow the drops to be absorbed into the agar, the plates were placed in incubators for 24 h at 37 °C, and then the plates were read (see Figure S8).

In addition, another assay was performed to compare the effect on the three bacteria of the more concentrated (NS_2_) nanosystem, synthesized as described in the previous section. The same protocol was used, replacing nanoparticles with the NS_2_ nanosystem. The results are shown in Figure S9.

#### 2.2.9. Preparation of Pellets for TEM and SEM Microscopes

Bacterial solutions were prepared for each bacteria (*E. coli*, *S. aureus* and *S. pneumoniae*) by placing several fresh (twenty-four hours’ growth) colonies in 40 mL of Mueller Hinton medium until a high turbidity was reached. Next, 1000 µL of bacterial dilution was transferred to six 2 mL Eppendorf tubes, and another 750 µL to six 1.5 mL Eppendorf tubes. Finally, the same volume (1000 µL in 2 mL Eppendorf tubes and 750 µL in 1.5 mL Eppendorf tubes) of the six compounds used in the microtiter plates, that is, nanoparticles 1 and 2, AMOX, Mueller Hinton (MH), and NS_1_ and NS_2_ nanosystems were added to each Eppendorf tube. The tubes were gently mixed and incubated for 24 h in an incubator at 37 °C.

After the incubation time, the Eppendorf tubes were gently agitated and then centrifuged at 3500 rpm for two minutes. Then, the supernatant was removed, and 200 µL of glutaraldehyde was added to each Eppendorf tube to fix the content. The tubes were gently agitated one more time and then remained motionless. After an hour, the tubes were centrifuged under the conditions previously described, the supernatant was removed and 500 µL of cacodylate, which is a washing solution, was added. After agitating for 10 min to re-suspend the pellet, the tubes were centrifuged again (3500 rpm for two minutes). Washing was repeated three times, leaving the supernatant of the final wash ready to be observed with TEM and SEM microscopes.

## 3. Results and Discussion

### 3.1. Au@16-mph-16/DNA-AMOX Complex Formation and Conformational Changes in DNA/AMOX Complexes Induced by Au@16-mph-16 Cationic Nanoparticles

**Figure 1:**
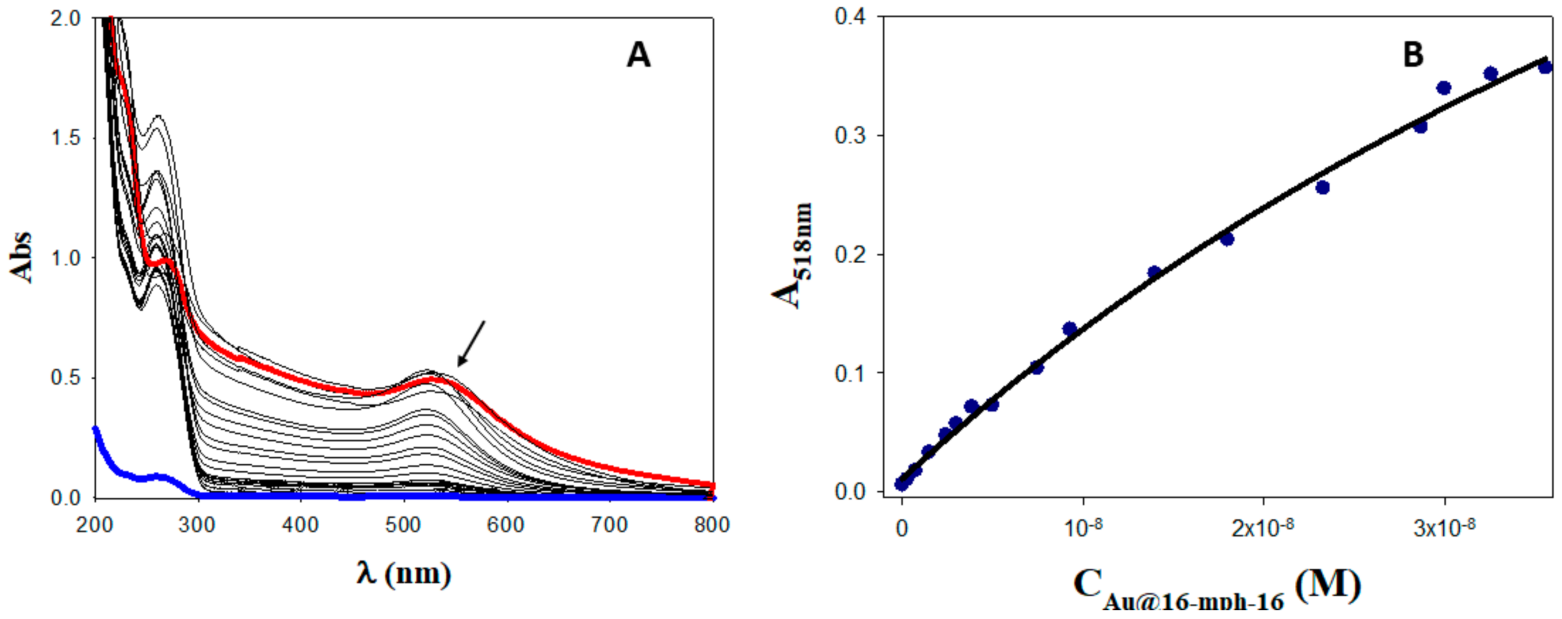
(**A**) Absorption spectra of the DNA/AMOX complex in the presence of different Au@16-mph-16 concentrations. Curves correspond from blue to red spectra to 0, 0.028, 0.075, 1.49, 2.44, 3.00, 3.86, 5.00, 5.70, 7.46, 9.30, 14.0, 18.0, 23.3, 28.7, 30.0, 31.6, 32.6, 33.6, 35.6, 37.6 and 40.9 nM of Au@16-mph-16. C_DNA_ = C_AMOX_ = 68 μM. (**B**) Analysis of the absorbance titration data for the DNA/AMOX-Au@16-mph-16 system in water. C_DNA_ = C_AMOX_ = 0.97 nM, λ = 518 nm, T = 298.2 K. Data are fitted according to a two-state model (Equation (3)).

As is already known, the use of nanocomplexes that include an antibiotic in their structure is an effective and efficient strategy to combat resistance to antimicrobials, while also minimizing side effects [21,49]. In this work, we used Au@16-mph-16/DNA-AMOX nanocomplexes to efficiently transport AMOX to the interior of the target. To obtain stable nanocomplexes with gemini surfactant-functionalized AuNPs (Au@16-mph-16) and AMOX, we employed DNA as a linker biomolecule. DNA is a genetic material with high biocompatibility and low cytotoxicity, making it ideal for applications in biomedicine [21,44,50,51,52]. Therefore, its use in the manufacturing of systems for the transport of antimicrobials to the interior of the target could help reduce antimicrobial resistance. One of the most convenient methods for detecting complex formations of DNA biopolymers and ligands is to monitor the changes in UV-visible spectra of the complex at different C_DNA_/C_Ligand_ mixing ratios, where C_DNA_ corresponds to the DNA concentration in base pairs and C_Ligand_, the ligand concentration [53]. Previous studies based on spectroscopic and voltametric techniques have shown that AMOX binds to CT-DNA via electrostatic and groove binding interactions with K = 2.7 × 10^4^ M^−1^ [43]. Thus, starting from the formation of the DNA/AMOX complex prepared under saturation conditions (see Section 2.2.2), we added increasing amounts of Au@16-mph-16 cationic nanoparticles to obtain stable Au@16-mph-16/DNA-AMOX complexes. Figure 1A shows the absorbance spectra of the Au@16-mph-16/DNA-AMOX system in water, where no well-defined isosbestic point is observed at about 540–547 nm, suggesting the presence of different binding modes and the complex nature of the interaction mechanism.

As shown in Figure 1A, with the addition of increasing amounts of AuNPs, there is an increase in the absorbance registered. This hyperchromic effect could be due to strong electrostatic and hydrophobic interactions between the nanoparticle and the ds-DNA, promoting breakage of the DNA structure [54]. However, according to Patel et al. the observed hyperchromic effect could be compatible with different binding modes such as intercalation, groove binding, or external binding [55]. Hence, additional structural studies are needed to distinguish them.

**Scheme 2:**
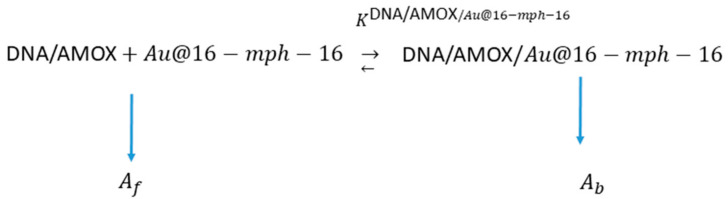
Schematic representation of the absorbance signal distribution according to the two-state model. A_f_ and A_b_ represent the absorbance of the free DNA/AMOX complex and the complex bound to the surface of AuNPs, respectively.

Based on changes in absorbance spectra as Au@16-mph-16 nanoparticles were added to the DNA/AMOX complex solution, it was possible to quantify the binding constant of the interaction between the DNA complex and the nanoparticles. According to the two-state model, changes in the absorbance registered at a fixed wavelength are the consequence of the distribution of the AuNPs in the bulk (water), and on the DNA/AMOX surface (see Scheme 2) [56].

According to the two-state model, if a complex is formed between the DNA linked to AMOX and Au@16-mph-16 nanoparticles, there will be two populations at the equilibrium of DNA/AMOX, one free and one bound to the nanoparticle:

(1) $${\left[\mathrm{DNA}/\mathrm{AMOX}\right]}_{f}=\frac{1}{1+K^{\mathrm{DNA}/\mathrm{AMOX}/\mathrm{Au}@16-\mathrm{mph}-16}\left[\mathrm{Au}@16-\mathrm{mph}-16\right]}{\left[\mathrm{DNA}/\mathrm{AMOX}\right]}_{0}$$

(2) $${\left[\mathrm{DNA}/\mathrm{AMOX}\right]}_{b}=\frac{K^{\mathrm{DNA}/\mathrm{AMOX}/\mathrm{Au}@16-\mathrm{mph}-16}\left[\mathrm{Au}@16-\mathrm{mph}-16\right]}{1+K^{\mathrm{DNA}/\mathrm{AMOX}/\mathrm{Au}@16-\mathrm{mph}-16}\left[\mathrm{Au}@16-\mathrm{mph}-16\right]}{\left[\mathrm{DNA}/\mathrm{AMOX}\right]}_{0}$$

where [DNA/AMOX]_0_ is the total concentration of DNA, assuming the complete formation of the DNA-AMOX complex [43], and [DNA/AMOX]_f_ and [DNA/AMOX]_b_ correspond to the DNA populations free and bound to the nanoparticle, respectively, such that [DNA]_0_ = [DNA/AMOX]_f_ + [DNA/AMOX]_b_. Accordingly, the measured absorbance at 518 nm may be given:

(3) $$A_{518\mathrm{nm}}=\frac{A_{f}+{\;A}_{b}K^{\mathrm{DNA}/\mathrm{AMOX}/\mathrm{Au}@16-\mathrm{mph}-16}\left[\mathrm{Au}@16-\mathrm{mph}-16\right]}{1+K^{\mathrm{DNA}/\mathrm{AMOX}/\mathrm{Au}@16-\mathrm{mph}-16}\left[\mathrm{Au}@16-\mathrm{mph}-16\right]}$$

Figure 1B gives the variation of A_518nm_ with varying gold nanoparticle concentrations, yielding the value of the equilibrium binding constant of the interaction. Thus, by fitting the experimental values of A_518nm_ to Equation (3), a value of K^DNA/AMOX/Au@16-mph-16^ = (1.2 ± 0.3) × 10^7^ M^−1^ was obtained in water. The high value of the apparent equilibrium binding constant highlights the tightness of binding, probably due to groove binding or partial intercalation [57]. However, additional structural studies are needed to assess the binding mode.

**Figure 2:**
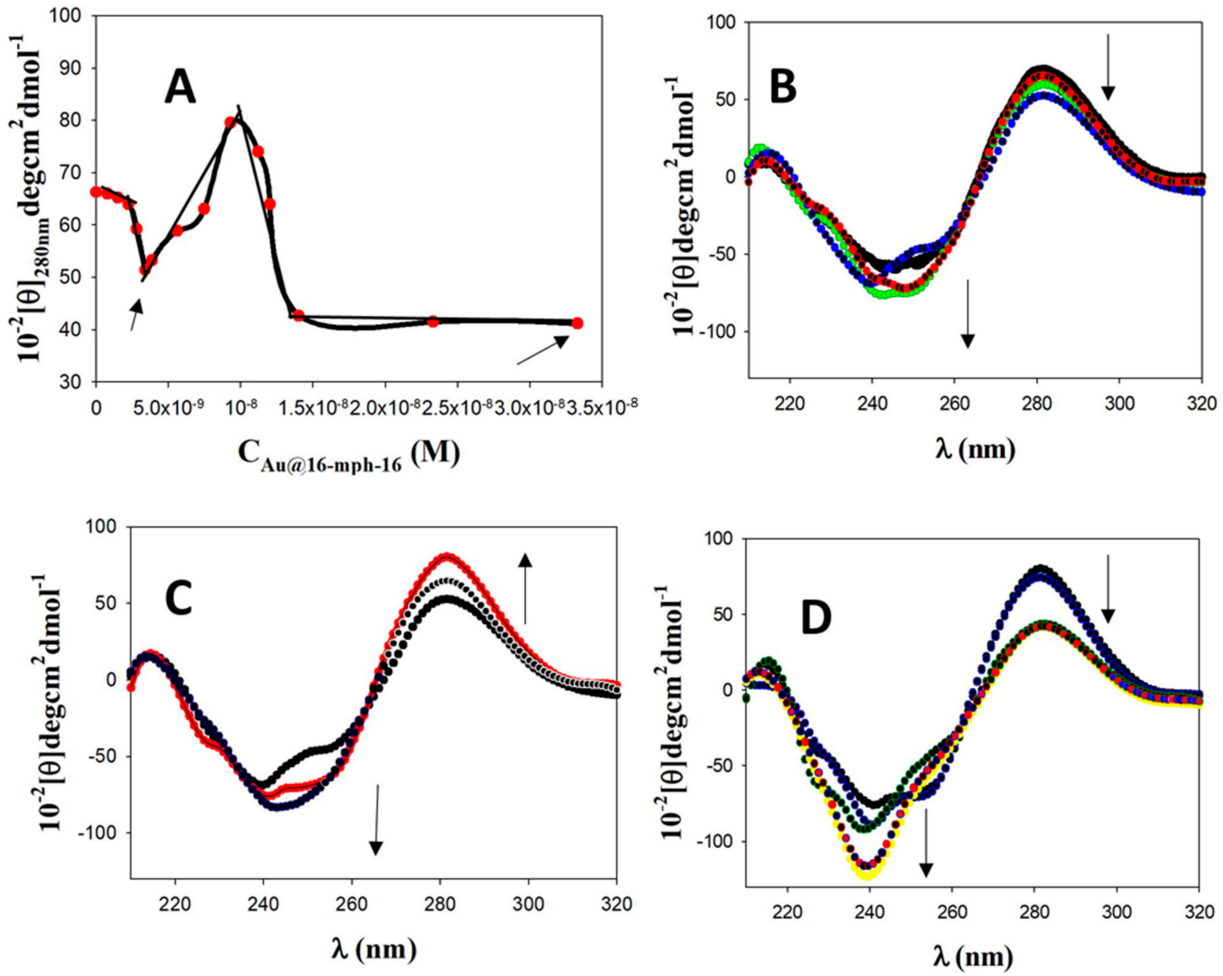
CD study for the interaction of Au@16-mph-16 with DNA/AMOX complex at different C_Au@16-mph-16_ concentrations. C_DNA_ = 68 μM and C_AMOX_ = 68 μM. T = 298.2 K. (**A**) CD trend in molar ellipticity units ([θ]_280nm_) at 280 nm. (**B**) Curves correspond to C_Au@16-mph-16_ values of (**B**) (●) 0, (●) 1.49 nM, (●) 2.80 nM and (●) 3.4 nM. (**C**) (●) 3.4 nM, (●) 7.46 nM and (●) 9.30 nM. (**D**) (●) 9.30 nM, (●) 11.2 nM, (●) 14.0 nM, (●) 23.0 nM and (●) 33.3 nM. The arrows show the direction of CD change.

In addition, the interaction of both the cationic gemini surfactant that constitutes the nanoparticles and the gold core with DNA foments the compaction of the biomolecule itself, facilitating entry to the bacterial interior in its compacted form [21,44,58]. Therefore, it is expected that disturbances will appear in the secondary structure of the DNA/AMOX complex in its interaction with AuNPs, which could modify the biochemical and biological effects of free AMOX. Figure S3 (in red) shows a CD spectrum of DNA in the right B form, showing the intensities of the similar negative and positive peaks at 280 nm and 249 nm. It is known that the intrinsic CD spectrum of DNA in the region from 210 to 320 nm is susceptible to modification by DNA interactions with ligands, producing stacking between DNA bases as well as changes in the helical superstructure of the polynucleotide [59]. The spectrum of free AMOX is recorded in blue. When AMOX was added to the DNA system in the absence of AuNPs (see Figure S3, in black), perturbations in the intensity of both bands were observed. However, as AMOX displays intrinsic CD spectra in the same region as DNA, the spectra of the DNA/AMOX complexes were corrected for the contribution of AMOX in each case (see Figure S3, in green). Figure 2 shows the changes in corrected CD spectra of the DNA/AMOX complex as a function of C_Au@16-mph-16_.

Different behavior was observed in Figure 2A, where the molar ellipticity of the positive band at 280 nm is plotted vs. C_Au@16-mph-16_, showing the appearance of two minima at 3.4 nM and 33 nM, respectively, and a maximum at 10 nM. As shown in Figure 2B, once the DNA/AMOX complex was formed, the subsequent addition of increasing concentrations of C_Au@16-mph-16_ to a fixed amount of DNA/AMOX complex progressively decreased the intensity of the positive CD band and increased the intensity of the negative band. Note that the same trend was registered again when higher concentrations of C_Au@16-mph-16_ were added after passing the maximum (see Figure 2D). This behavior is indicative of DNA denaturalization and double helix unwinding; these features are compatible with partial intercalation processes and DNA compaction [60,61]. On the other hand, the CD trend registered in Figure 2C shows how the intensities of both CD bands are enhanced without modifying their positions. This behavior is compatible with the disruption of the stacking contact of the DNA bases necessary for Au@16-mph-16 partial intercalation [40,62]. Note that considerable untwisting of the DNA helical backbone, destacking, and destabilization of the bases are needed to accommodate the nanoparticles into the DNA base pairs and overcome possible steric bulk. Thus, we postulate that the compacted nanocomplexes formed at the two minima are essentially different in structure. That is, the nanocomplex formed at the first minimum is a more labile external complex than that formed after passing the maxima, in which AuNPs are previously intercalated into the DNA/AMOX complex.

Hence, considering the two minima positions at 3.4 nM and 33 nM, we prepared Au@16-mph-16/DNA-AMOX complexes, designated as NS_1_ and NS_2_ nanosystems (see Section 2.1.4) to study the effect of these structurally distinct nanocarriers on the bactericidal properties of AMOX.

### 3.2. Stability, Charge, and Size of Au@16-mph-16 and Au@ 16-mph-16/DNA-AMOX Nanosystems and Release Kinetics

**Figure 3:**
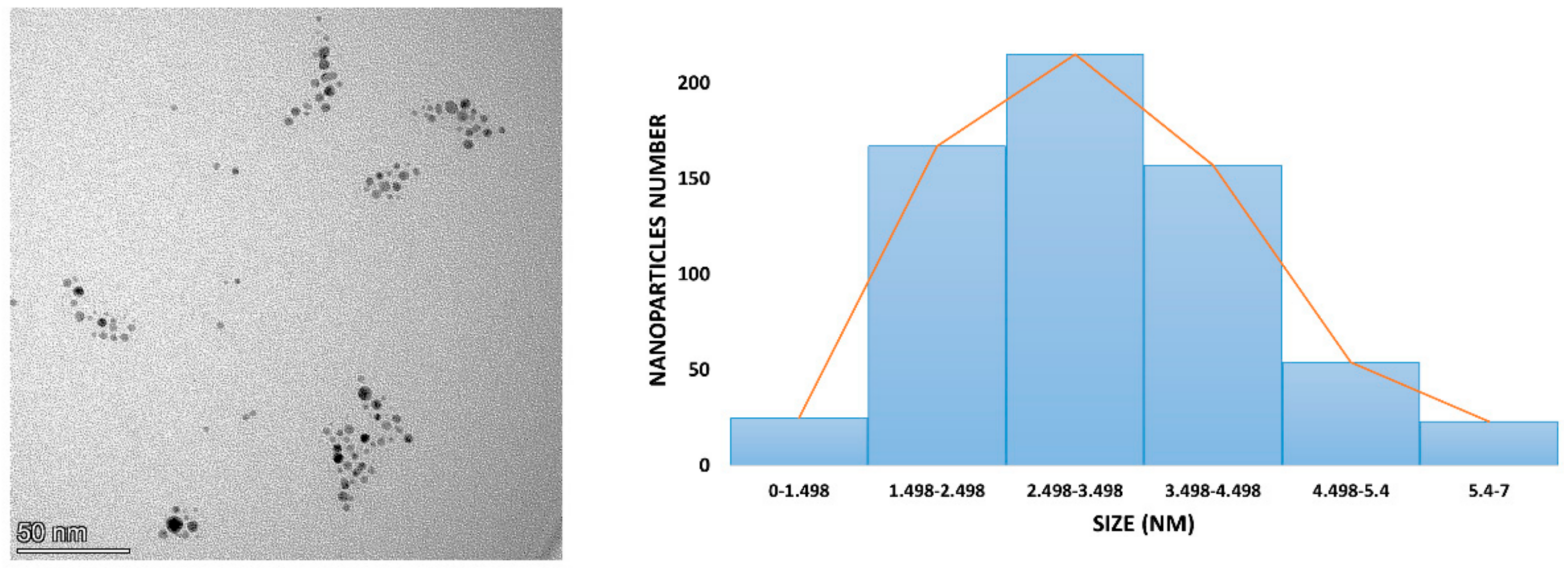
Microphotograph made with TEM TALOS, showing the gold core of the nanoparticles and the corresponding histogram of size distribution on 350 gold cores of the nanoparticles, produced using ImageJ software.

**Figure 4:**
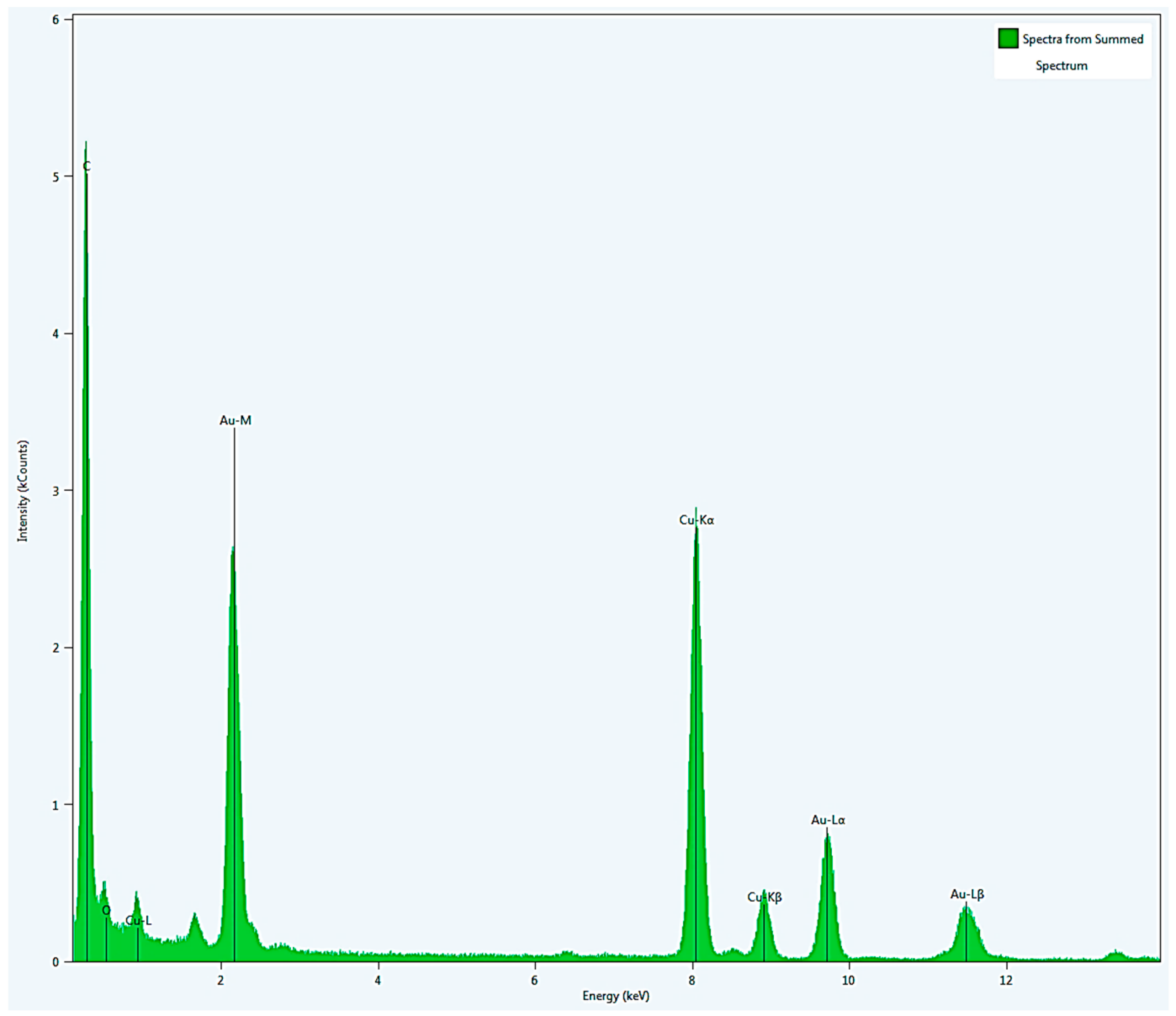
EDS-MET graph showing the chemical composition of Au@16-mph-16 gold core nanoparticles. Inorganic compounds such as Copper (Cu) and Gold (Au) were observed.

To analyze the size of the AuNPs with precision, a study was made with TEM (Figure 3), showing a spherical shape and a size of (3.2 ± 0.9) nm. The study was completed with EDS-MET microanalysis, verifying the presence of gold in the sample studied (Figure 4). Results show that gold is present; other elements, such as copper, also appear, and form part of the grid wherein the ultra-thin sections were deposited for study.

**Figure 5:**
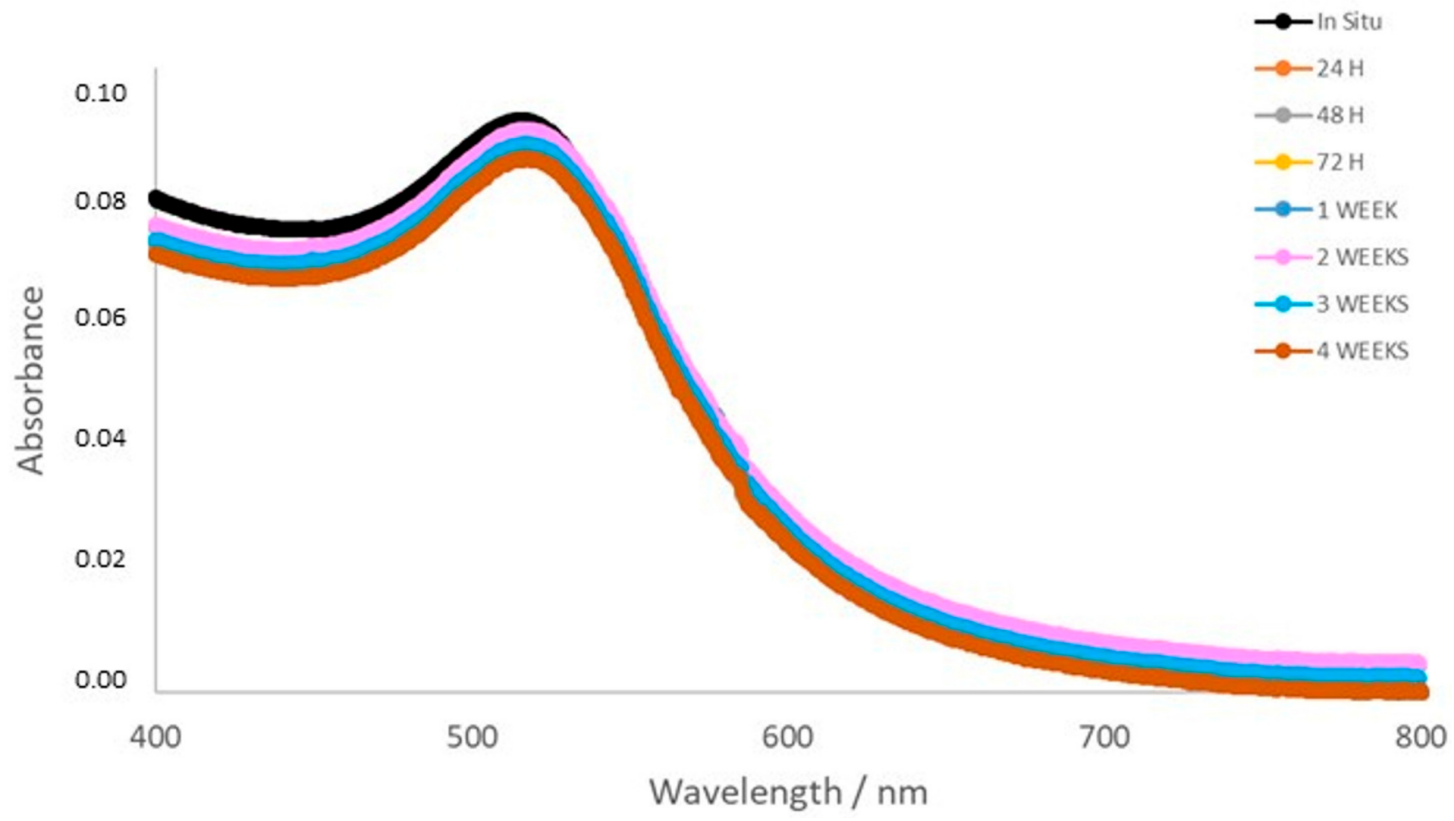
Stability study over time, performed using UV-visible spectrophotometry, for Au@16-mph-16 nanoparticles.

**Figure 6:**
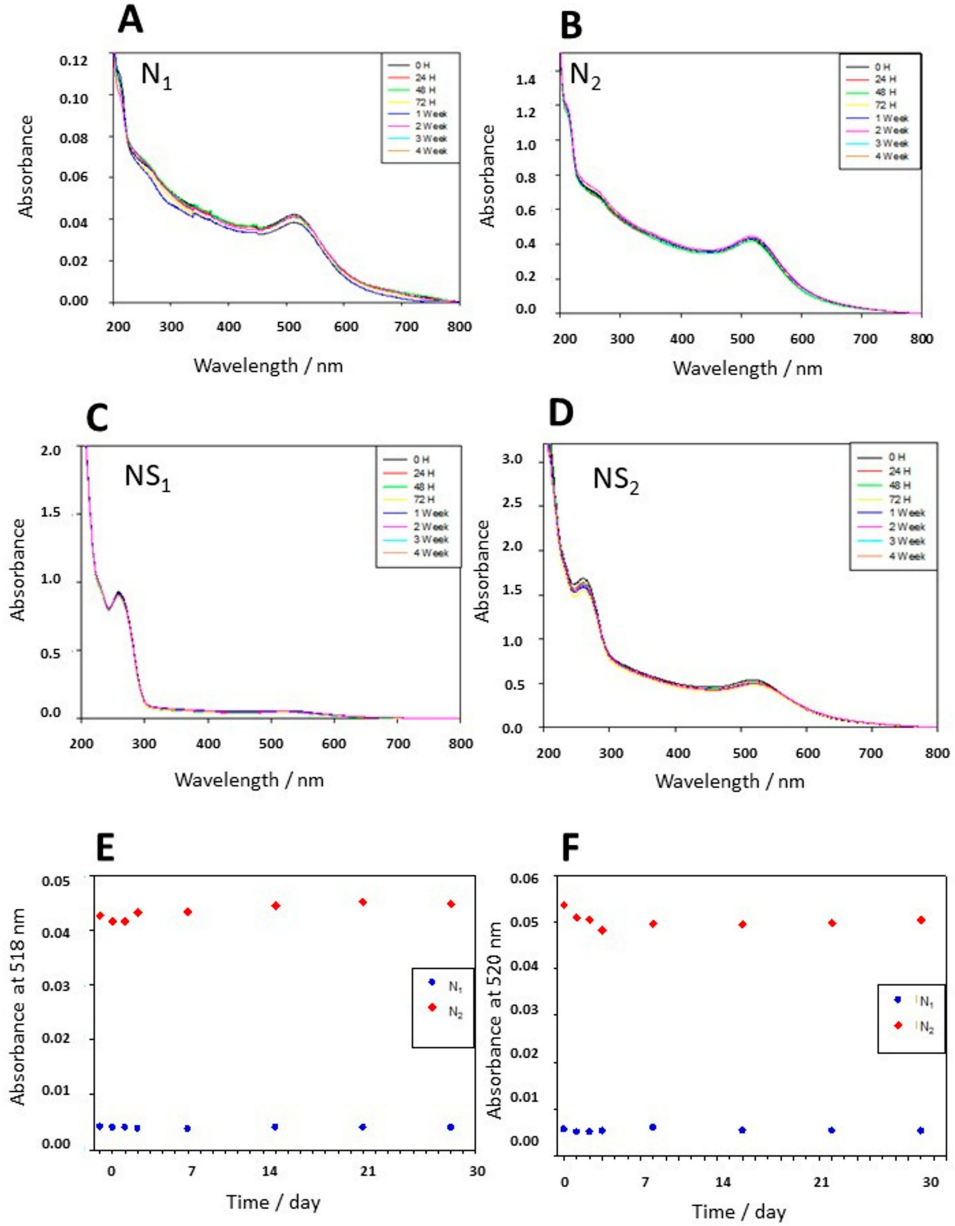
Absorbance spectra and evolution over time of different nanosystems. (**A**,**B**) Absorbance spectra of N_1_ and N_2_ (Au@16-mph-16 precursors). (**C**,**D**) Absorbance spectra of NS_1_ and NS_2_ (Au@16-mph-16/ADN/AMOX) compacted nanosystems. (**E**) Evolution over time of absorbance at fixed 518 nm wavelength (SPR location) for Au@16-mpPh-16 precursors at different concentrations; blue triangles correspond to [Au@16-mph-16] = N_1_, and red diamonds correspond to [Au@16-mph-16] = N_2_. (**F**) Evolution over time of absorbance at a fixed 520 nm wavelength for compacted nanocomplexes at different concentrations; blue triangles correspond to [Au@16-mph-16/DNA/AMOX] = NS_1_ and red diamonds correspond to [Au@16-mph-16/DNA–AMOX] = NS_2_.

One of the most important qualities of nanoparticles to be used as drug carriers is their stability. Therefore, the stability of different Au@16-mph-16 and Au@16-mph-16/DNA-AMOX formulations was studied using UV-vis spectrophotometry. Figure 5 shows the UV-visible spectra of the Au@16-mph-16 nanoparticles, wherein the absorbance curves are practically superimposed, indicating their stability over one month. As can be seen, the figure does not show evidence of the SPR peak broadening; thus, possible significant effects of aggregation on nanoparticles are ruled out [63]. Figure 6 shows the stability of N_i_ (Figure 6A), N_2_ (Figure 6B), NS_1_ (Figure 6C) and NS_2_ (Figure 6D) over a month, demonstrating high stability in the periods studied. On the other hand, the evolution in time of the absorbance at a fixed wavelength of 518 nm (SPR location) (Figure 6E) was verified for the Au@16-opPh-16 precursors at different concentrations and points in time. With the absorbance at a fixed wavelength of 520 nm for compacted nanocomplexes at different concentrations, these results demonstrate the stability of both the precursors and the nanosystems (Figure 6F).

Characterization analyses, in addition to measuring size and charge, are effective in measuring their stability when dispersed in a specific solvent. The zeta potential that represents the total charge on the nanoparticle surface, being a high positive or negative charge of around ±30 mV, is considered optimal to achieve physical colloidal stability [64].

As shown in Table 1, the zeta potential values of the different systems studied are higher than the range mentioned above for stability. Thus, optimal stability is guaranteed. A well-defined zeta potential peak is observed for all the samples studied, as can be seen in Figure S5. The zeta potential of the Au@16-mph-16 nanoparticles is highly positive in relation to the charge of the surfactant gemini micelles that stabilize the nanosystem. In contrast to this, the charge of the DNA/AMOX complex is highly negative, since negatively charged phosphate groups constitute the polymer backbone.

Table 1 and Figure S4 also show the hydrodynamic size of Au@16-mph-16/DNA-AMOX complexes in which a bimodal distribution of their size is evidenced for NS_1_ nanocomplex. The largest size population, which is in a clearly lower percentage, can be identified with the existence of some nanocomplexes in a more extended conformation. Thus, the smallest size population can be attributed to the formation of compacted nanostructures. Note that the percentage of compacted nanostructures is higher than 95%, revealing that the degree of compaction of the NS_1_ nanocomplex is quite high. In the case of the NS_2_ nanosystem, the compaction is fully accomplished, as can be observed in the unimodal size distribution for this complex, which has a mean size of 69 nm.

**Figure 7:**
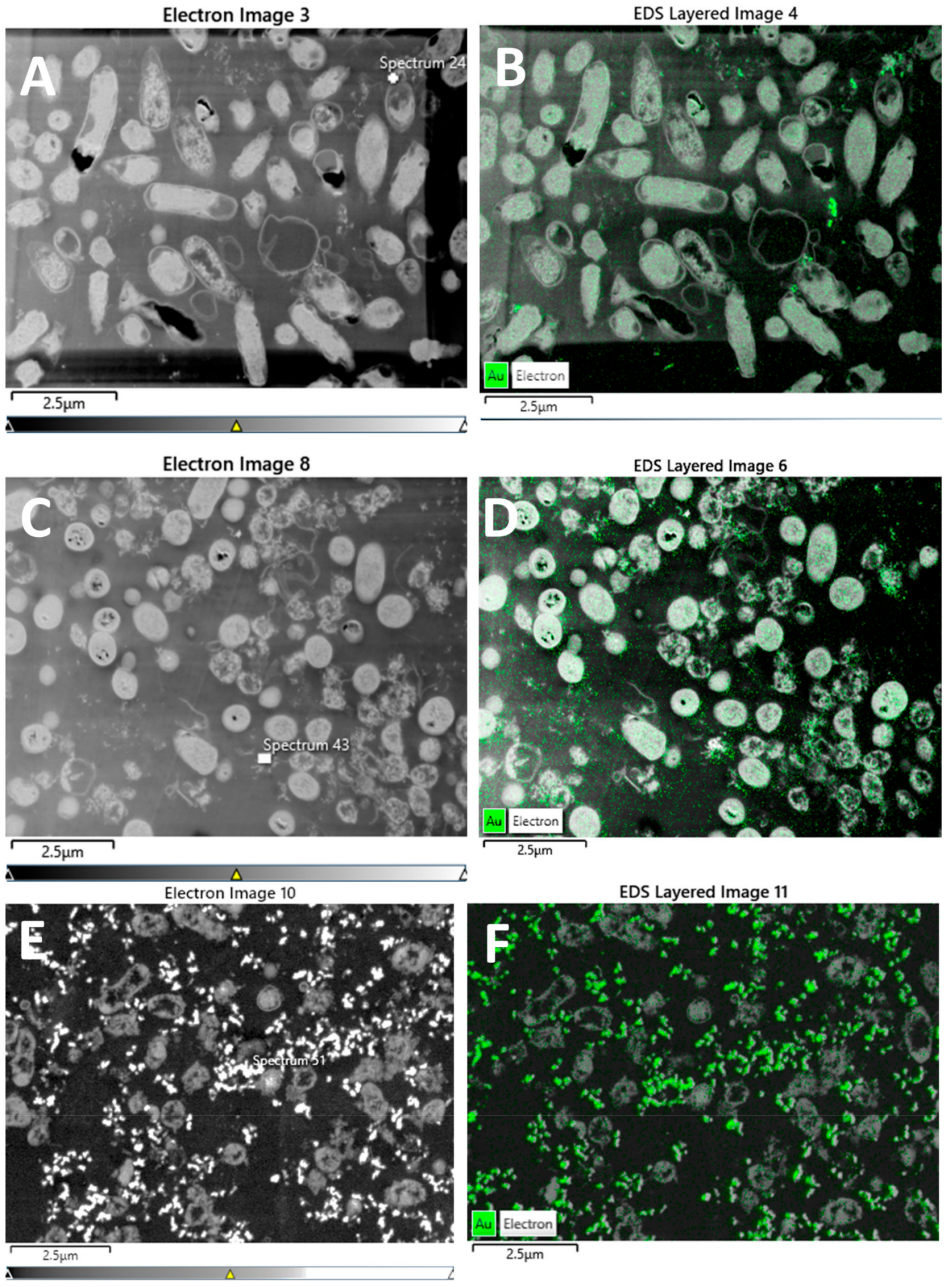
SEM images of different populations of bacteria: (**A**) *E. coli*, (**C**) *S. aureus* and (**E**) *S. pneumoniae*. SEM-EDS analysis of the chemical analysis of the sample showing the presence of Au from the nanoparticles: (**B**) *E. coli*, (**D**) *S. aureus* and (**F**) *S. pneumoniae*.

**Figure 8:**
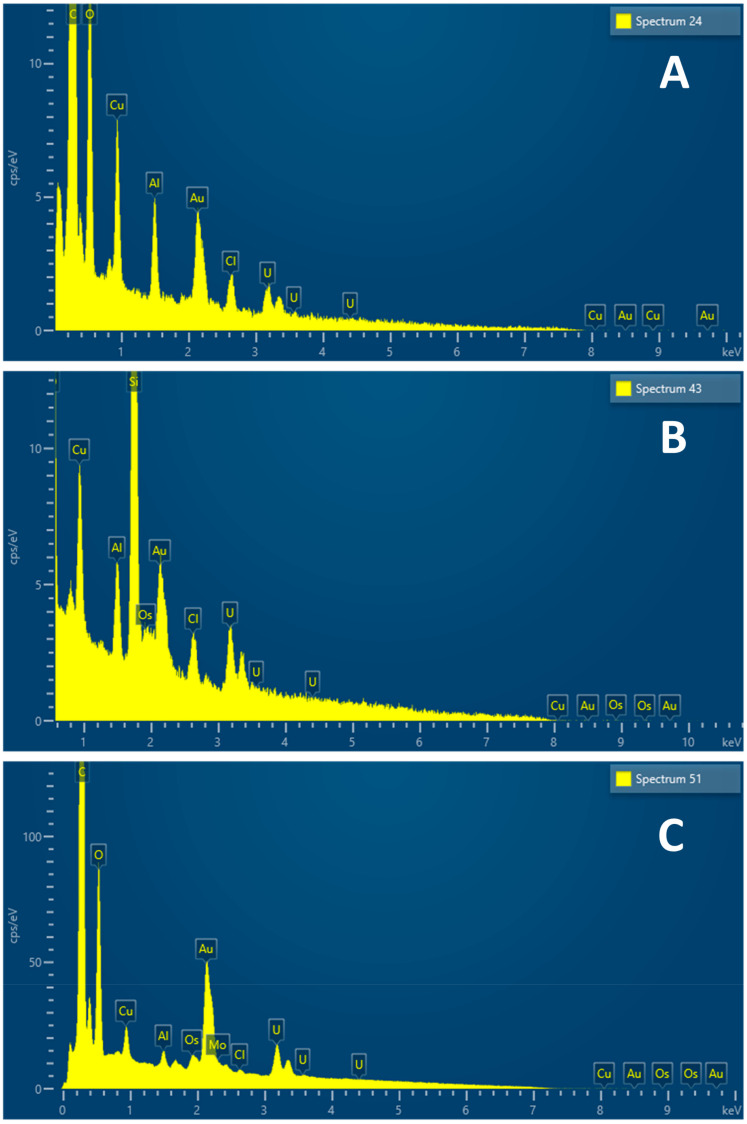
Spectrum produced to verify the presence of gold inside the microorganisms (**A**) *E. coli*, (**B**) *S. aureus,* and (**C**) *S. pneumoniae*. Note that elements such as the constituents of microorganisms appear alongside some used in the preparations.

Since only inorganic elements can be visualized in the TEM Talos, given that the high voltage destroys organic elements, we carried out an EDS study with an SEM that allowed microanalysis to be carried out inside microorganisms without destroying organic elements. In the fine-tuning of the material, tests were carried out on specific slides and a copper grid. For both preparations, it was necessary to determine the most suitable thickness of cut, which was determined to be 150 nm. The studies were carried out with N_2_ in all cases. Figure 7A shows the electronic image for *E. coli*, Figure 7C for *S. aureus,* and Figure 7E for *S. pneumoniae*. Figure 7B (*E coli*), Figure 7D (*S. aureus*), and Figure 7F (*S. pneumoniae*) show the EDS microanalysis on the bacteria sample, and Figure 8 shows the spectrum produced to verify the presence of gold inside microorganisms. Note that elements such as the constituents of the microorganisms appear alongside some used in the preparations.

Finally, it is important to note that any antibiotic nanocarrier depends on a reduction in the required dosage to be effective, and an increase in the circulation half-time in order to combat multidrug-resistant bacteria [65]. Thus, studying the release kinetics of drug delivery systems would help in optimizing the dosage regimen and ensuring optimal drug concentration at the target site, this being particularly important when a free drug has poor pharmacokinetics/biodistribution [66]. In this sense, the release kinetics of the nanoparticles play a vital role in determining the duration and effectiveness of the therapeutic action of both nanosystems. Thus, in this study, the release kinetics were investigated using a UV-visible technique in Mueller Hinton (MH) media at pH = 7.3 and at 37 °C to mimic the bacterial environment. To achieve this, changes in the absorbance at 520 and 230 nm wavelengths were followed with the time course of the reaction (see Figure S6). The goodness-of-fit of the release data was initially tested by employing distinct kinetics models such as zero-order and first-order kinetics models, the Hixson–Crowell cube–root model, and the Higuchi model (see Figure S7). However, for all these models, except for the Hixson–Crowell cube–root model, the calculated correlation coefficient was lower than 0.78. In the case of the Hixson model, despite the fit of the kinetic data being statistically highly significant (R^2^ = 0.901), the calculated lag times were negative and unreasonable (t = −363 s), considering the duration of the kinetic experiment. Thus, all these tested models were discarded for understanding the release profiles of AMOX from the nanocomplexes. Because the release data seemed to follow a biphasic model profile, the goodness of the fit to a bi-exponential first-order kinetic model (Equation (4)) was also tested [67]:

(4) $$w=a\times e^{-k1x}+b\times e^{-k2x}$$

**Figure 9:**
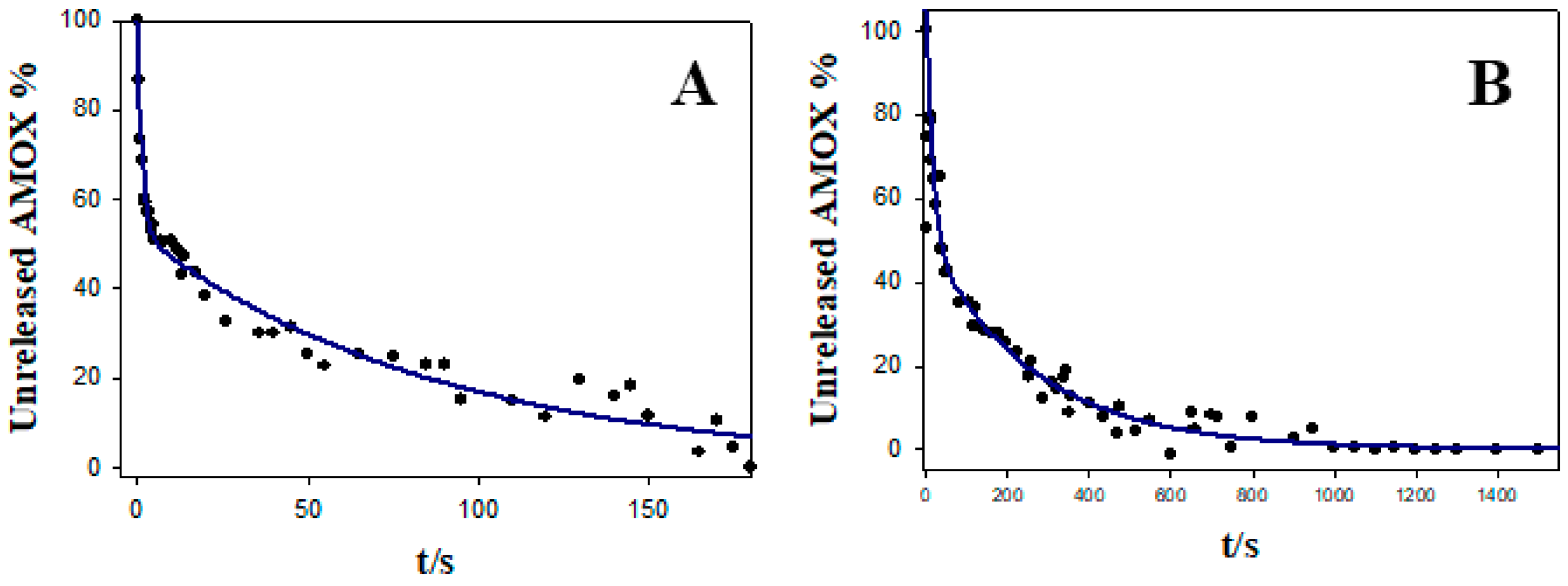
Unreleased amounts of AMOX (%) from the nanocomplexes vs. time in Mueller Hinton medium at 37 °C. The continuous line corresponds to the best fit obtained with the bi-exponential first-order kinetic model described in Equation (4). (**A**) NS_1_ formulation, (**B**) NS_2_ formulation.

Detailed calculations are demonstrated in Figure 9 and Table 2. In this equation, w is the unreleased amount of AMOX at time t, and k_1_ and k_2_ are the release rate constants of the initial phase and terminal phase, respectively. Lag time was defined as the calculated value of t corresponding to w = 100%.

From the obtained results, it can be concluded that the fast initial phase of the AMOX release kinetic, which is determined by a k_1_ rate constant, can mainly be attributed to the destabilization and disintegration of the nanosystem in the bacterial environment. This entails a fast enhancement in the dissolution area for the drug. The terminal phase, which is controlled by the k_2_ kinetic constant, mainly describes the increase in the dissolution of the AMOX in the media after the nanocomplex disintegration. On the other hand, the release kinetic of NS_1_ is one order of magnitude faster than that of the NS_2_ complex. This fact can be explained considering the high charge of the NS_2_ nanosystem that confers them high stability in different media. As the AMOX concentration is fixed in both nanoformulations, we can conclude that drug release kinetics can be tuned by adjusting nanoparticle charge, size, and concentration in the nanocomplex. Note that these results are promising for some antibiotic therapies, such as those related to acute infections, prophylaxis, or the management of endogenous diseases, in which a fast systemic distribution is needed [68].

### 3.3. Results of Gold Nanosystems’ Susceptibility Tests against Gram+ and Gram− Reference Strains

**Figure 10:**
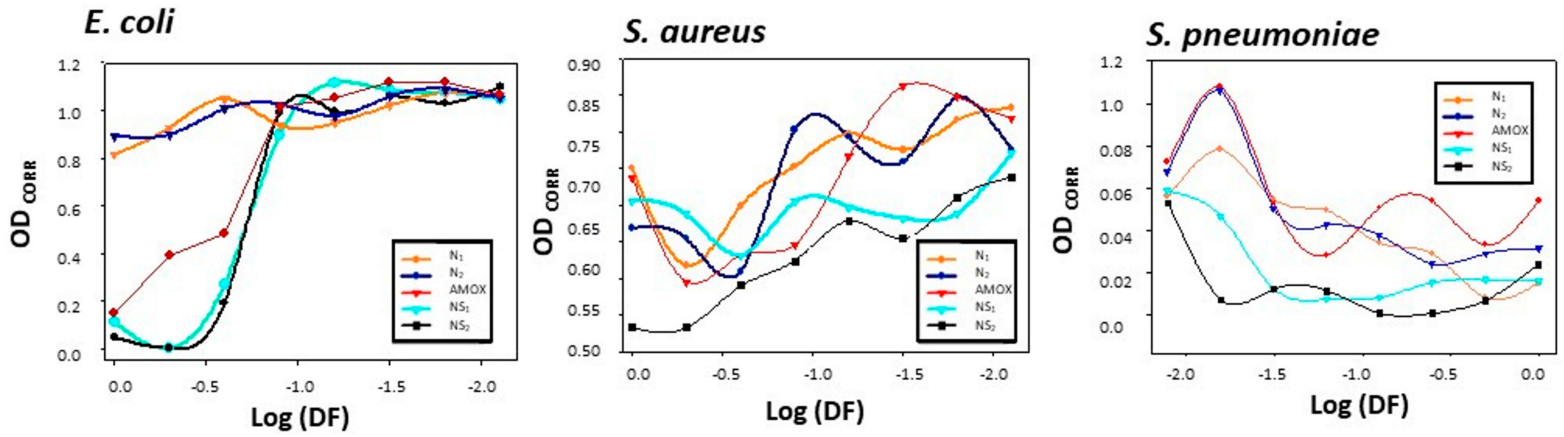
Microdilution assay. Spectrophotometry results for distinct bacterial agents. Dots correspond to experimental data, and solid lines to trend lines. AMOX control is shown in red, N_1_ and N_2_ AuNPs in orange and in blue, respectively, and NS_1_ and NS_2_ nanosystems in black and in cyan, respectively. OD_CORR_ corresponds to the corrected optical density of the systems (see Section 2.2.8 for more details).

The Au@16-mph-16 precursors and the compacted Au@16-mph-16/DNA-AMOX nanosystems obtained were tested at two different concentrations in each case on Gram-positive and Gram-negative bacteria, as a viability study to demonstrate their effectiveness and antimicrobial effects. A plate assay of nanoparticles and nanosystems was conducted, and the effects of the more concentrated nanoparticle (NP_2_; 32.6 nM) and nanosystem (NS_2_; 32.6 nM), their ½ and ¼ dilutions, and a commercial AMOX disk (25 µg) were evaluated (Figure S8). Neither AMOX nor the nanoparticle exhibited a visible effect on *S. aureus* or *E. coli,* as shown in Figure 10, while the effect of the nanoparticle on *S. pneumoniae* was poor. However, AMOX had a great inhibitory effect on *S. pneumoniae* (Figures S9 and S10).

The nanosystem had a marked effect on Gram-positive bacteria, while not having a visible effect on *E. coli*. A decrease in inhibitory effect with the dilution of the nanosystem was observed for *S. aureus.*

Although *S. aureus* and *S. pneumoniae* are both Gram-positive rods, there are two structural/biochemical differences between the two species which may be the cause of the differences in the effect of the gold nanoparticles/nanosystem. First, *S. pneumoniae* possesses a polysaccharide capsule, which minimizes or inhibits recognition by the host, protecting the pneumococcus against phagocytic clearance by blocking the deposition of immunoglobulins (Ig) and inhibition of complement the pneumococcal cell surface [69]. However, we consider that these differences can be attributed to a greater extent to the presence in *S. aureus* of the catalase enzyme, which enhances virulence potential due to its ability to evade killing by neutrophils [69]. Neutrophils act by producing reactive oxygen species (ROS), while nanoparticles and nanosystems act via a similar biosynthetic machinery disruption mechanism, creating numerous ROS [21,70,71,72,73,74,75,76,77]. Thus, in both cases, they act by inactivating the catalase enzyme, which may result in a greater effect of the nanoparticles on *S. pneumoniae* compared to *S. aureus*.

On the other hand, the results on *E. coli* are not surprising, due to the limited effect of beta-lactam antibiotics on Gram-negative bacteria [78].

The results of the microdilution plate assay can be observed in Figure 10 and Figure S10. The macroscopic results from the microdilution plates show the minimal inhibitory concentrations (Table 3). Again, there are great variations according to species. In this sense, the growth of *S. pneumoniae* was very poor in the plates (no buttons observed) because of its greater nutritive requirements compared with non-exigent bacteria of genus *Staphylococcus* or *Escherichia*. To maintain the same conditions in the assay, Mueller Hinton broth was used, but this is not the best choice for bacteria belonging to genus *Streptococcus* [79]. On the other hand, it can be appreciated that there was no inhibition effect on *S. aureus* (bacterial buttons were observed at the bottom of all wells), so MIC could not be estimated. However, button size clearly decreased with dilution in most of the rows. In the case of *E. coli*, NP_2_ had a mild effect, but only when not diluted (Table 3). The effect of NS_1_ and NS_2_ was greater, reaching an MIC value of 17 µM.

In addition, the optical density of wells in microdilution plates was read via UV-visible spectrophotometry. The results, once corrected by detracting the optical density of the wells in the control plate and after logistic transformation, are shown in Figure 10. The *E. coli* chart shows a good inhibitory effect of both nanosystems and AMOX in a non-diluted and half-diluted form (columns 1 and 2), but it disappears for all compounds in more diluted wells. The *S. aureus* chart shows a greater inhibition effect of NS_2_ and NP_2_ in non-diluted wells. With dilution, the effect progressively decreases (and optical density increases) for AMOX, nanoparticles, and nanosystems, with nanosystems exhibiting the greater effect (especially the more concentrated nanosystem, NS_2_). Finally, the poor growth of *S. pneumoniae* made the spectrophotometry results more erratic, but we can still appreciate that optical density was higher in more diluted wells (8th column), with a greater effect shown for nanosystems (i.e., a lower optical density).

### 3.4. Internalization of Au@16-mph-16 and Au@16-mph-16/DNA-AMOX Nanosystems

After these studies were carried out, treatments were performed using both the precursor and the most concentrated nanosystems, N_2_ and NS_2_, respectively. In addition, controls without treatment (with MH) and treatment only with AMOX were considered. TEM photomicrographs were taken with low contrast to highlight the gold nuclei that form both nanoparticles and nanosystems, thus distinguishing them from other dense organelles such as ribosomes.

**Scheme 3:**
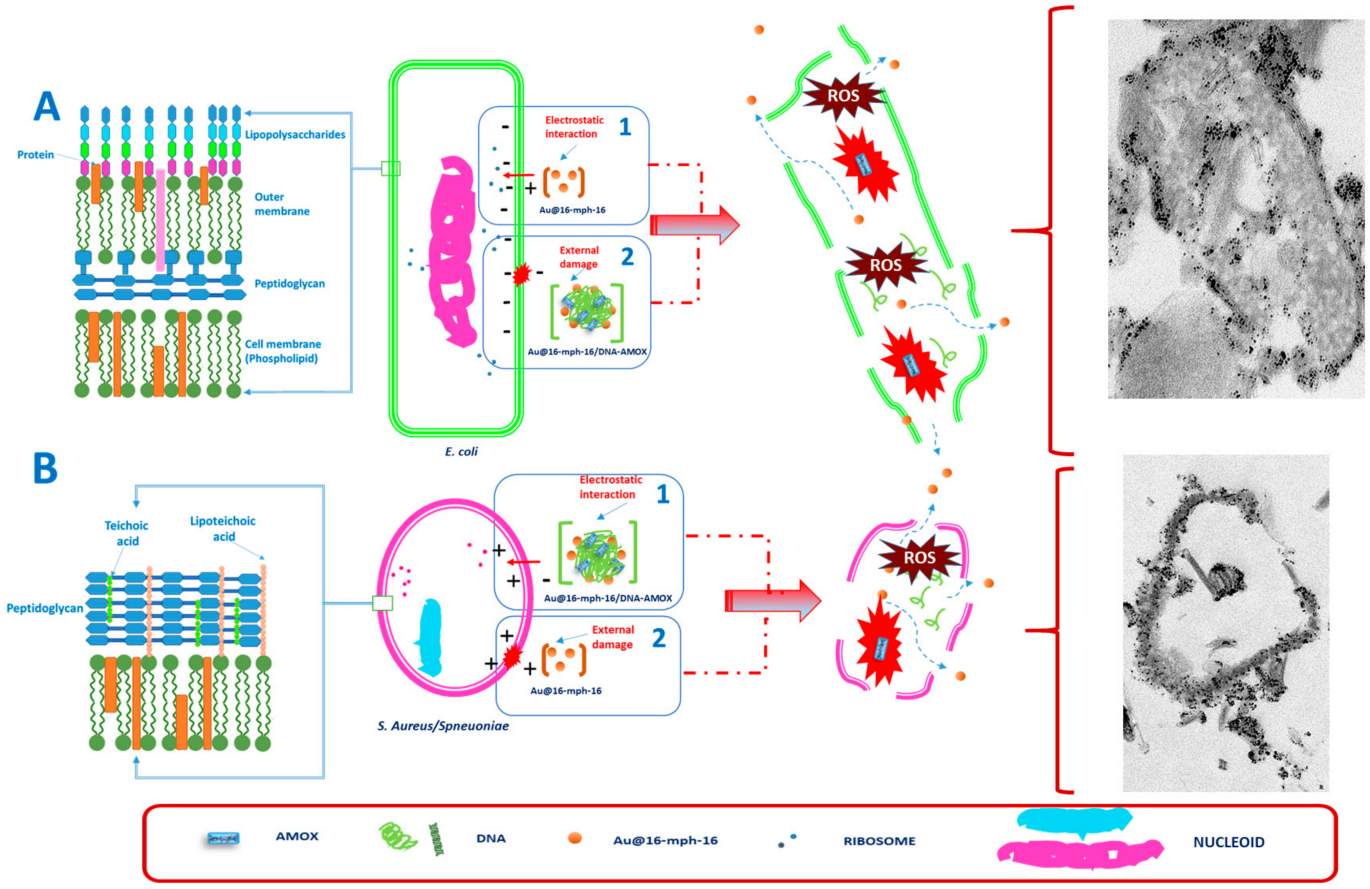
Illustration of the possible mechanisms of action of Ni precursors (Au@16-mph-16) and NSi nanosystems (Au@16-mph-16/DNA-AMOX) on Gram-negative (**A**) and Gram-positive bacteria (**B**). On the left of the image, the differences between the walls of Gram-negative and Gram-positive bacteria are illustrated.

Gram-negative bacteria are surrounded by an outer membrane and immersed in a periplasmic space (Scheme 3A), and Gram-positive bacteria are immersed in an aniconic matrix of sugary polymers (Scheme 3B). In the case of Gram-negative cells, the precursors (Ni) are introduced into the microorganism by endocytosis, due to their electrostatic attraction (part 1 of Scheme 3A). In the case of nanosystems (NSi), damage is produced on the surface of the bacteria, which allows it to penetrate inside, releasing the antibiotic (part 1 of Scheme 3A). In both cases, once inside, the antibiotic is released from the precursor and causes a mismatch in the transduction of metabolic signals, destroying the biosynthetic machinery of the microorganism and causing its death [21,70,71,72,73,74,75,76,77]; it is the production of numerous free radicals (which are impossible for the microorganism to neutralize) which contributes to the destruction of microorganisms, without time to generate resistance. In the case of Gram-positives (Scheme 3B), the internalization of the nanosystem, as illustrated in part 1 of Scheme 3B, occurs via electrostatic interactions, while in the case of the precursors (part 2 of Scheme 3B), superficial damage is produced, which makes it easy for the microorganisms to penetrate inside. In both cases, the production of free radicals and the interruption of the biosynthetic machinery cause the bacteria to be destroyed.

**Figure 11:**
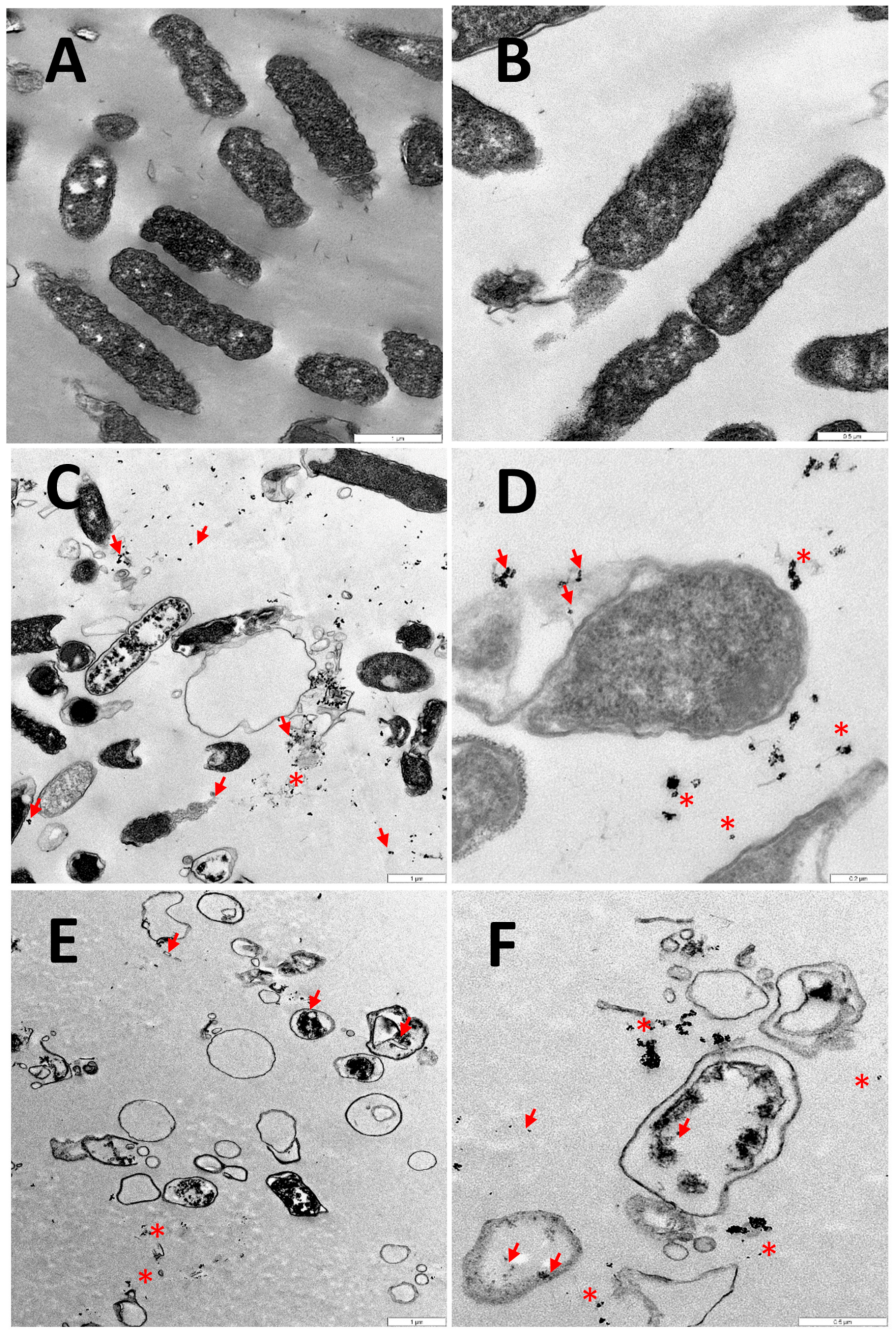
TEM microphotograph of *E. coli* after 24 h of treatment. (**A**) Control bacteria without treatment. (**B**) Bacteria treated with AMOX. (**C**,**D**) Bacteria treated with the N_2_ precursor. (**E**,**F**) Bacteria treated with the NS_2_ nanosystem. Arrows indicate the presence of metallic nuclei compatible with the gold cores of the nanoparticles. Asterisks (*) indicate the remains of nanoparticles surrounded by biological material because of the action of the nanosystems.

Figure 11 shows the effects of the different treatments on cultures of *E. coli*, a Gram-negative bacterium. Figure 11A shows a control population with MH only. The results of treatment with AMOX are shown in Figure 11B; some bacterial affectation in the integrity of the bacterium surface can be observed. Figure 11C,D show the results of the application of the N_2_ precursor; affectation in a greater number of bacteria can be observed. This mechanism of action may be due to electrostatic attraction, since N_2_ is strongly positive; thus, endocytic internalization may occur. Once inside, the mechanism of action for the interruption of the biosynthetic machinery is similar to that described previously [21,70,71,72,73,74,75,76,77]. However, in the case of treatment with the NS_2_ nanosystem (Figure 11E,F), penetration into the interior and subsequent action is produced by surface destabilization, since the nanosystem has a negative charge.

**Figure 12:**
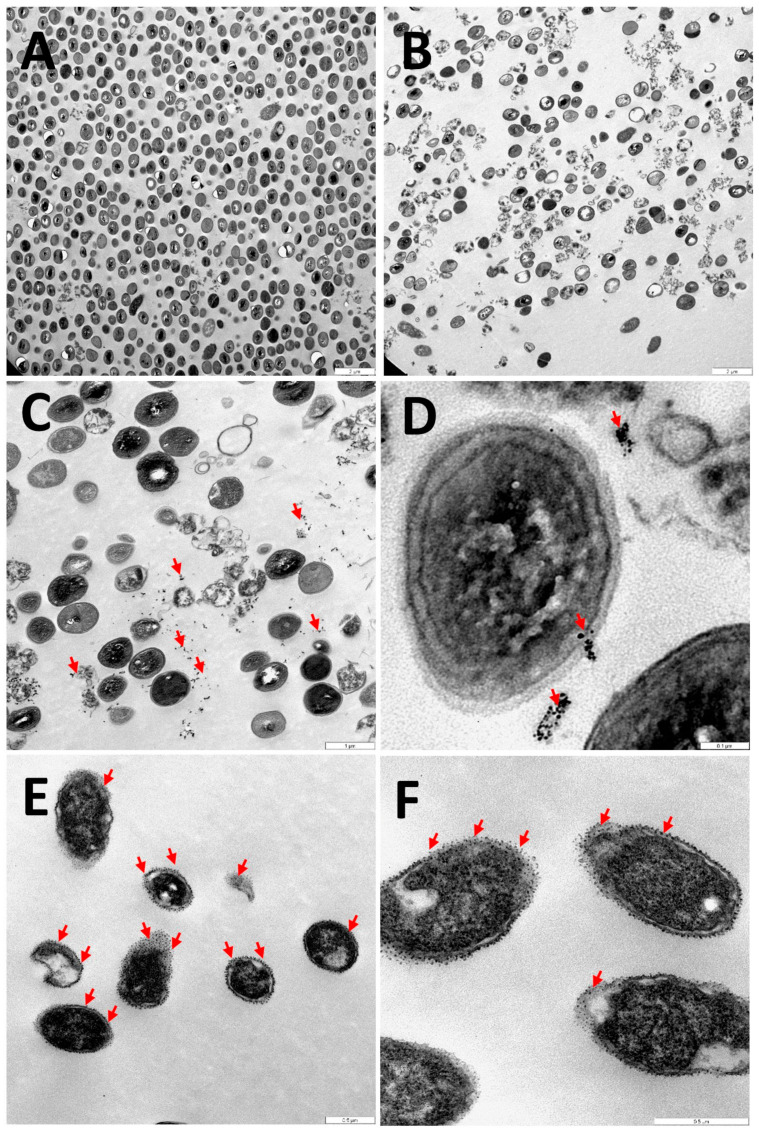
TEM photomicrograph of S. aureus after 24 h of treatment. In (**A**), the control is shown, corresponding to a population of bacteria without treatment. In (**B**), populations of bacteria treated only with the antibiotic (AMOX) are observed. The treatment with the precursors is shown in (**C**,**D**). The population of bacteria treated with NS_2_ is shown in (**E**,**F**). The arrow indicates the presence of metallic nuclei compatible with the gold nuclei of the nanoparticles.

Figure 12 shows the results obtained in a culture of *S. aureus* at 24 h. In Figure 12B, the effect of AMOX on the population of bacteria can be seen; some elements have lost structural integrity, and some have been destroyed. Figure 12C,D show the results of treatment with the nanosystem precursor. There is an effect similar to that previously observed with the AMOX treatment. AuNPs possess strong antibacterial properties for Gram-negative and Gram-positive bacteria [21,80,81,82,83]. For this reason, bacteria are affected by N_2_ treatments. Since these bacteria are Gram-positive, and N_2_ has a positive charge, N_2_ may produce structural damage at the surface level; subsequently, the destabilization of the structure allows the nanoparticles to penetrate the bacteria, producing an increase in oxidative stress. Consequently, there will be a dysfunction in the protein and enzymatic metabolic pathways, causing an inhibition of metabolic signal transduction and therefore, causing the death of the bacteria [21,70,71,72,73,74,75,76,77]. In Figure 12E,F, we can see the effect of the treatment with the nanosystem. Since the NS_2_ charge is negative, the mechanism of action is driven by an electrostatic interaction, contributing to internalization by endocytosis, releasing the antibiotic from the precursor and causing a mismatch in metabolic signal transduction similar to that described above, thereby destroying the biosynthetic machinery of the microorganism and causing its death [21,70,71,72,73,74,75,76,77].

**Figure 13:**
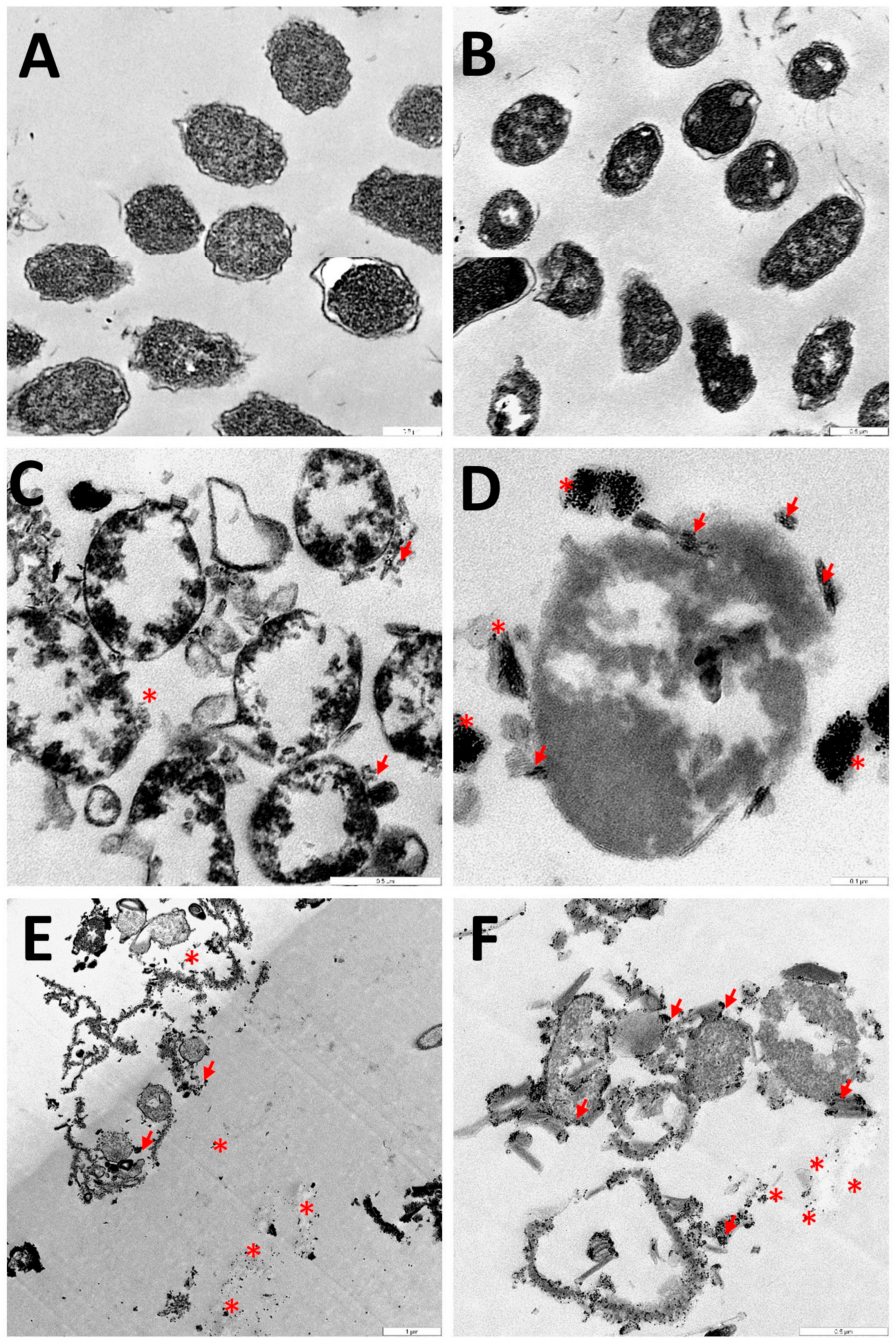
TEM microphotograph corresponding to a population of *S. pneumoniae* after 24 h of treatment. (**A**) Control bacteria without treatment. (**B**) Bacteria treated with the AMOX antibiotic. (**C**,**D**) Bacteria treated with the N_2_ precursor. (**E**,**F**) Bacteria treated with the NS_2_ nanosystem. Arrows indicate the presence of metallic nuclei compatible with the gold cores of the nanoparticles. Asterisks (*) indicate the remains of nanoparticles surrounded by biological material because of the action of the nanosystems.

Finally, in Figure 13, we can see the result of the treatments carried out on *S. pneumoniae*, which is a lactic acid, facultative anaerobic, catalase-negative bacterium. In Figure 13A, the normal population in a culture with MH is shown. In Figure 13B, little bacterial involvement is observed because of AMOX. In Figure 13C,D, we see a strong effect of the treatment with the precursor, and in Figure 13F,G, we see extreme devastation with the application of the nanosystem, which causes a potentiation of the antibiotic effect in these bacteria. It is important to note that the NS_2_ preparation has the same AMOX concentration as the control in Figure 13B; thus, the results obtained here support the benefit of using Au@16-mph-16 nanoparticles as nanocarriers for antibacterial drugs. Being a Gram-positive bacterium, the destabilization mechanisms of the biosynthetic machinery via the induction of the release of free oxygen radicals are similar to those previously described for *S. aureus* [21,70,71,72,73,74,75,76,77]. However, these bacteria are not reactive to catalase, while *S. aureus* are positive. Catalase is one of the enzymes involved in the destruction of hydrogen peroxide generated during metabolism [84]. Therefore, the action of the precursors and the nanosystems causes the damage to be greater in *S. pneumoniae* than in *S. aureus*, in which the antioxidant protection system that participates in the transformation of said reactive oxygen species slows down the mechanism of performance, although it does not prevent it.

Once the nanoparticle characterization of the nanosystem and their study with Gram+ and Gram− reference strains have been accomplished, a comment about the effect of Au@16-mph-16 concentration on the antibacterial effect of the formulation seems to be pertinent. As we know, nanoparticle concentration has an important effect on physicochemical properties such as surface charge, size, composition and aggregation state of the nanoformulations [85,86,87]. A proof of concept is given in our study with varying Au@16-mph-16 concentrations in NS_1_ and NS_2_ nanosystems which produce gold nanocarriers with different size and charge (see Table 1). Moreover, distinct studies have demonstrated that nanomaterials can act as antibacterial complements to antibiotics; they are highly promising, and can act synergistically by filling the gaps wherein antibiotics frequently fail [88]. Thus, changing the nanoparticle concentration in Au@16-mph-16/DNA-AMOX nanosystems could affect the antibacterial effect of a given nanoformulation, as its physicochemical properties will be substantially modified. In fact, current research has shown that the size of metal nanoparticles can greatly affect their antibacterial activity; smaller nanoparticles with larger specific surface areas for interaction with the bacterial cell membrane can internalize more efficiently than those with larger size, thus increasing their antibacterial activity [89]. However, the size is not the dominant factor to be controlled in assessing the antibacterial properties of a nanosystem, as has previously been demonstrated by Deplanche and coworkers [90]. In this study, the antibacterial activity of three types of Mg(OH)_2_ nanoparticle varying in size against *E. coli* mutant strains has been tested. However, the results showed that the smallest Mg(OH)_2_ nanoparticle had the weakest antibacterial effect. Thus, careful attention should be paid to the values of other physicochemical properties such as zeta potential. In fact, current studies have revealed that the zeta potential value in a nanosystem may strongly affect bacterial adhesion [89]. Thus, the nature of the electrostatic interaction between the negatively charged bacterial cell membrane and the surface charge of the nanosystem could enhance or diminish their interaction. In our study, it has been demonstrated that both NS_1_ and NS_2_ nanoparticles internalize efficiently into the bacterial wall. However, the antibacterial effect of NS_2_ is higher than in the case of NS_1_, as demonstrated by bacterial culture studies (see Figure 10). Thus, the unimodal size distribution and smaller size of the NS_2_ nanosystem, as well as the high gold concentration in the formulation, enhance its antibacterial effect. However, as NS_2_ nanosystems have a more negative zeta potential than NS_1_ formulations (−45.7 vs. −36.7 mV), their high stability in solvent media and poor bacterial adhesion could influence their release kinetics, being one order of magnitude slower than in the case of NS_1_ (see Table 2). Moreover, the effect of gold nanoparticle concentration should also be discussed in the case of naked Au@16-mph-16 nanoparticles N_1_ and N_2_. As they are of a smaller size in comparison with NSi nanosystems, and have a highly positive charge (+67.8 mV), electrostatic attraction with the bacterial wall is guaranteed. Moreover, the antibacterial activity and internalization of the more concentrated N_2_ is greater than in the case of N_1_, as has been demonstrated by susceptibility testing against different bacterial strains (see Figure 10). Thus, in this work, it has been demonstrated that the nanoparticles’ concentration and their physicochemical properties are key factors to be controlled and optimized to obtain efficient vectors for combating antibiotic resistance.

Despite the promising results obtained in this study and in different research on similar MBPs-antibiotic/biopolymer nanomaterials [25,26,27,28,29,30,31,32,33], there are still some problems. due to these materials’ own nature, that need to be overcome and further investigated. One of the principal problems is related to the toxicity of nanoparticles coupled with antibiotics. The study of this research gap is fundamental to producing safe products for commercial approval by the U.S. Food and Drug Administration (FDA) [91,92]. In this sense, nanomaterials that uses small AuNPs as metal-based nanoparticles in their structure are promising, because they appear to be harmless; this is because they are inert in nature, and non-toxic at low gold concentrations. In fact, these nanosystems can enter, establish themselves in the cell via the pinocytosis pathway, and localize to lysosomes without entering the core (all of this in conjunction contributing to minimizing their hazardousness) [93]. In addition, the redox nature of gold is beneficial in reducing the level of reactive oxygen produced during exposure to nanoparticles. For instance, in several studies of a histological nature, after prolonged treatment in rats, it was established that AuNPs are not toxic at concentrations of 0.25, 0.5 or 1 mg/kg to the brain, liver, kidneys, heart, spleen or lungs [94]. Other toxicity studies of AuNPs coupled to mRNA, after 7 days of treatment in mice, showed that there are no residues in the main vital organs [45]. In this sense, the explored Au@16-mph-16/DNA-AMOX nanoformulations are promising because they are small-sized (69 nm in the case of NS_2_) and have low concentrations of gold metal. However, the possible impact and potential of these nanocarriers and other similar ones must continue to be verified to avoid adverse effects in organisms, and to develop innocuous delivery systems without losing efficacy. All together, these efforts will contribute to increase the long-term safety profiles of these nanomaterials [95]. Another important issue to be controlled and improved for possible commercial and medical applications is related to the stability of MBPs–antibiotic/biopolymer nanomaterials. In this sense, the goal of the work is the high stability of the NS_1_ and NS_2_ nanosystems in water; they are stable for at least one month due to their great charge in solution.

Thus, the resulting smaller sized Au@16-mph-16/DNA-AMOX nanocomplexes act both by generating an oxidative stress response and interacting with the cellular membrane, inducing a much stronger antibacterial effect and ensuring biocompatibility. Thus, the gold precursors and the configured Au@16-mph-16/DNA-AMOX nanosystems act quickly, favoring microbial death with a small amount of antibiotic and combating resistance to antibiotics in addition to avoid secondary side effects derived from the administration of high doses of antibiotics. In spite of the promising results provided by different researchers of MBNPs-antibiotic/biopolymer nanomaterials, there still are some important problems to be solved in relation to storage times, stability, and toxicity associated with the studied nanoformulations. In this sense, novel Au@16-mph-16/DNA-AMOX nanocomplexes have proved to be highly stable in aqueous media for at least one month, as demonstrated following the UV-visible spectra evolution of the complexes with time. Moreover, the stability of the nanocomplex has also been demonstrated by quantifying the equilibrium binding constant of the nanocomplex formation (K^DNA/AMOX/Au@16-mph-16^ = (1.2 ± 0.3) × 10^7^ M^−1^). Thus, Au@16-mph-16/DNA-AMOX are promising nanosystems able to produce safe and stable products for possible commercial applications. Finally, internalization experiments carried out using TEM microscopy have demonstrated that Au@16-mph-16/DNA-AMOX are promising nanocarriers for AMOX administration.

## 4. Conclusions

The high stability, low toxicity and good biocompatibility of AuNPs afford them high efficacy as vehicles for drug delivery; the dose of a given drug can be adjusted to the needs of patients with various pathologies. This fact is fundamental in the administration of antibiotics, since the adjustment of the dose, together with the penetration capacity in the target, can prevent the development of antibiotic resistance in microorganisms. In addition, the use of low doses may contribute to minimizing possible side effects. Both the AuNPs and the nanosystems used in this work show high stability over time, as demonstrated in spectrophotometric studies. Likewise, in the case of nanosystems, their stability is strengthened with the use of gemini surfactants as stabilizing agents. On the other hand, the high positive charge of the nanoparticle allows a favorable electrostatic interaction with the DNA/AMOX complex, and confers the capacity to induce DNA compaction. The binding of the DNA/AMOX complex to Au@16-mph-16 nanoparticles is highly favorable, as proven by the high value of the equilibrium binding constant K^DNA/AMOX/Au@16-mph-16^ = (1.2 ± 0.3) × 10^7^ M^−1^, obtained following the change in the absorbance of the SPR band. CD experiments evidenced DNA compaction and the formation of two nanocomplexes NS_1_ and NS_2_, stabilized by external binding and partial intercalation, respectively.

AMOX has a hydroxyl group that makes it more soluble in lipids and therefore gives it greater bioavailability, duration of action, and bactericidal activity. AMOX is normally administered orally, and it is usually rapidly absorbed, presenting greater bioavailability. However, the dose of the antibiotic must be adjusted, since various side effects may present. TEM and SEM microscopy studies allowed us to identify gold in the sample composition, and allowed us to determine the average of the size of our nanoparticle and observe its morphology and internalization. For all these reasons, the results obtained in this work show that the nanosystems composed of the Au@16-mph-16 nanoparticles and the DNA/AMOX complex are appropriate for medical use. In addition, Au@16-mph-16 nanoparticles have been shown in studies to have strong antibacterial properties for both Gram-negative and Gram-positive bacteria. Thus, a synergistic effect is obtained by using AMOX and AuNPs in conjunction. Therefore, the possibility of administering them to patients with a wide range of bacterial infections, with a significant reduction in side effects, is evident, given that a greater effect was observed in the case of nanosystems, and a more equivalent effect was observed on bacteria treated with AMOX and nanoparticles. In particular, the action of the nanosystem on *S. pneumoniae* caused great destruction of the bacterial population; its use is possibly a good strategy for treating respiratory tract infections or meningitis, among other infections caused by this microorganism.

Thus, in light of the results obtained using different techniques, we have proved the synergistic effect of Au@16-mph-16 and AMOX against distinct bacterial strains when it is delivered as part of a Au@16-mph-16/DNA-AMOX (NSi) nanosystem. Thus, even at low Au@16-mph-16 concentrations in a nanosystem, the complexed antibiotic showed superior antimicrobial action compared to the equivalent doses of the free drug. Moreover, AMOX showed fast kinetic release in both nanosystems, which constitutes a promising result for its possible application in the treatment of acute infections or endogenous diseases. Although the studies carried out in this work on finding more effective therapies to combat resistance to antibiotics show promising results for highly resistant bacteria, it is advisable that we continue testing whether this efficacy can be transferred to other nanosystems coupled with different antibiotics. Moreover, these nanosystems must be tested for their efficacy on other resistant bacteria. In addition, in future work, their effects in complex organisms could be assessed in order to identify possible long-term adverse effects.

## Figures and Tables

**Table 1:** Zeta potential and hydrodynamic size expressed in size distribution by number for DNA/AMOX complex, Au@16-mph-16 nanoparticles, and Au@16-mph-16/DNA-AMOX nanosystems (NS_1_ and NS_2_). Abbreviations: d1 and d2 correspond to the mean diameters of the complexes that present a bimodal size distribution.

Sample	Sample Composition	Zeta Potential (mV)	Size (nm); Population %
1	Au@16-mph-16	67.8 ± 2.6	(2.6 ± 0.3)
2	DNA/AMOX C_DNA_ = 6.8 × 10^−6^ M/C_AMOX_ = 6.8 × 10^−6^ M	−73.9 ± 1.1	d_1_ = (85 ± 16.4); 6.8% d_2_ = (416 ± 42.9); 93.2%
3	NS_1_ (C_Au@16-mph-16_ = 3.4 nM)	−36.7 ± 1.0	d_1_ = (44 ± 8); 95.5% d_2_ = (205 ± 52); 4.5%
4	NS_2_ (C_Au@16-mph-16_ = 32.6 nM )	−45.1± 1.1	(69.0 ± 1.0)

**Table 2:** Bi-exponential first-order equations for the release of AMOX in Mueller Hinton medium at 37 °C.

Formulation	Equation	Lag. Time (s)	Correlation Coefficient (R^2^)
NS_1_	$w=47.4\times e^{-0.706t}+52.6\times e^{-0.0114t}$	0	0.977
NS_2_	$w=49.0\times e^{-0.0574t}+51.0\times e^{-0.0038t}$	0	0.920

**Table 3:** Minimum inhibitory concentration (MIC) values of antibiotics in the three types of bacteria studied.

	*E. coli*	*S. aureus*	*S. pneumoniae*
NP_1_	-	-	0.53
NP_2_	68	-	0.53
AMOX	34	-	0.53
Mueller Hinton media	-	-	0.53
NS_1_	17	-	0.53
NS_2_	17	-	0.53

## Data Availability

Data are contained within the article or Supplementary Materials.

## Supplementary Materials

The following supporting information can be downloaded at: https://www.mdpi.com/article/10.3390/antibiotics12081275/s1. Figure S1: bacterial growth in blood Mueller Hinton agar. Figure S2: Nuclear Magnetic Resonance (NMR) spectra and chemical structure of the 16-mph-16 gemini surfactant compound used for the formation of Au@16-mph-16 nanoparticles. Figure S3: CD spectra of DNA, AMOX and DNA/AMOX complex showing the correction made due to AMOX contribution in the intrinsic CD region. Figure S4: Dynamic Light Scattering (DLS) size distribution by number of Au@16-mph-16, DNA/AMOX complex and Au@16-mph-16/DNA-AMOX nanosystems in water. Figure S5: Results of measuring the Zeta potential of Au@16-mph-16, DNA/AMOX complex and Au@16-mph-16/DNA-AMOX nanosystems in water. Figure S6: Example of release kinetics profiles of AMOX from the nanosystems NS_1_ and NS_2_ obtained using a UV-visible technique in Mueller Hinton media at 37 °C. Figure S7: Curve fit of the kinetic data to distinct kinetic models and the corresponding correlation coefficients. Figure S8: The effect of an amoxicillin disk (25 µg) and nanoparticles (non-diluted and ½–¼ dilution) on bacterial cultures. Figure S9: The effect of an amoxicillin disk (25 µg) and nanosystem (non-diluted and ½–¼ dilution) on bacterial cultures. Figure S10: Microdilution plate assays with MIC results for *E. coli*, *S. aureus*, *S. pneumoniae,* and control using Mueller Hinton media.
