# Supplementary material for: Synergistic Antibacterial Effects of Amoxicillin and Gold Nanoparticles: A Therapeutic Option to Combat Antibiotic Resistance
Source: Antibiotics (Basel). 2023 Aug 2;12(8):1275. doi: 10.3390/antibiotics12081275 (PMC10451730; doi:10.3390/antibiotics12081275)
Supplement: Supplementary file 1 [file antibiotics-12-01275-s001.zip › antibiotics-2478396-supplementary.pdf]

Synergistic Antibacterial Effects of Amoxicillin and Gold Nanoparticles: A Therapeutic Option to Combat Antibiotic Resistance

Rosa M. Giráldez-Pérez [1,*,†], Elia M. Grueso [2,*,†], Alfonso Carbonero [3], Juan Álvarez Márquez [1], Mirian Gordillo [3], Edyta Kuliszewska [4] and Rafael Prado-Gotor [2]

[1] Department of Cell Biology, Physiology and Immunology, Faculty of Sciences, University of Cordoba, 14014 Cordoba, Spain; b82almaj@uco.es
[2] Department of Physical Chemistry, Faculty of Chemistry, University of Seville, 41012 Seville, Spain; pradogotor@us.es
[3] Department of Animal Health, Veterinary Faculty, University of Cordoba, 14014 Cordoba, Spain; sa1camaa@uco.es (A.C.); v82gomam@uco.es (M.G.)
[4] Chemtra Company, 47-300 Krapkowize, Poland
* Correspondence: rgiraldez@uco.es (R.M.G.-P.); elia@us.es (E.M.G.)
† These authors contributed equally to this work.

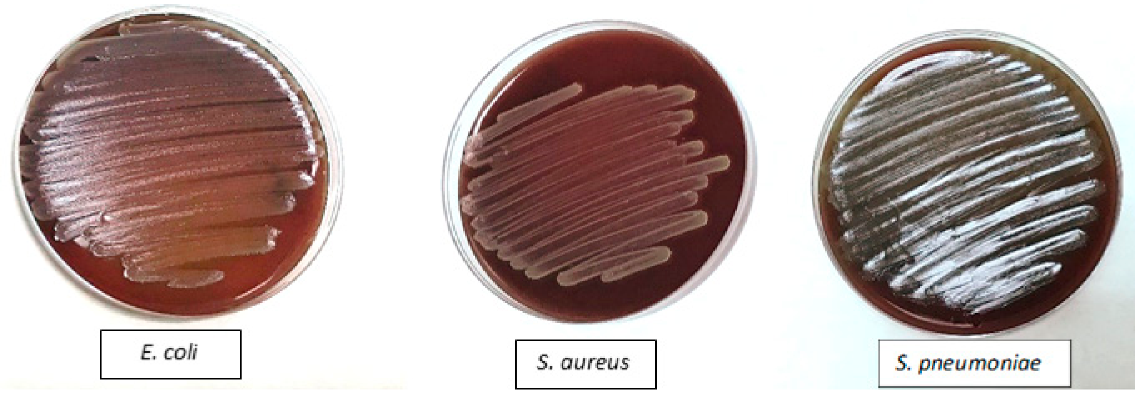

Figure S1. Bacterial growth in Blood Müller Hinton Agar

NMR Characterization of m-16-Ph-16 gemini surfactant

For NMR spectroscopic measurements, the 16-mph-16 compound (see Fig. S2 for the structure) was dissolved in 99.95% CDCl3 (~10 mg in 0.7 mL) and transferred into 5 mm NMR sample tubes (Promochem, Wesel, Germany). Spectra were measured on a Bruker DRX400 AVANCE spectrometer at 400.13 MHz (1H) or 100.62 MHz (13C) using the Topspin 1.3 (Bruker, Rheinstetten, Germany). For 2D spectra 32k data points were recorded and Fourier transformed to spectra with a range of 15 ppm (1H) and 240 ppm (13C). Two-dimensional COSY, TOCSY, NOESY, HMQC and HMBC spectra were measured with 128 experimental runs, each having 1024 data points each. Appropriate linear forward prediction, sinusoidal multiplication and

Fourier transformation led to 2D-spectra with ranges of 12 ppm and 220 ppm for 1H and 13C, respectively. Residual CHCl3 was used as internal standard for 1H (δH 7.24) and CDCl3 for 13C (δC 77.0) spectra. Measurement temperature was 298.1 K +/-0.1 K.

N,N′-Di-n-hexadecyl-N,N,N′,N′-tetramethyl-phenylene-1,3-dimethylenammonium dibromide (m-16-Ph-16). 1H-NMR (400 MHz, CDCl3, δ in ppm): 8.45 (s,br, 1 H, H-4); 7.76 (d,br, 2 H, J=6.4 Hz, H-2 (2x)); 7.45 (t,br, 1 H, J=6.4 Hz, H-3); 4.99 (s,br, 4 H, H-1′ (2x)); 3.47 (s,br, 4 H, H-1′′ (2x)); 3.20 (s,br, 12 H, CH3 (4x)); 1.79 (s,br, 4 H, H-2′′ (2x)); 1.32 (s,br, 4 H, H-3′′ (2x)); 1.24c (m,br, 48 H, H-4′′ to H-15′′ (2x)); 0.85 (t, 6H, J=7.0 Hz, H-16′′ (2x)). 13C NMR (100 MHz, CDCl3, δ in ppm): 138.8 (d, C-4); 135.1 (d, C-2 (2x)); 129.9 (d, C-3); 128.8 (s, C-1); 66.9 (t, C-1′ (2x)); 65.0 (t, C-1′′ (2x)); 49.9 (q, CH3 (4x)); 31.3 (t, C-14′′ (2x)); 29.7-29.4 (t (10x), C-4′′ to C-13′′ (2x)); 29.3 (t, C-3′′ (2x)); 26.4 (t, C-2′′ (2x)); 23.3 (t, C-15′′ (2x)); 14.1 (q, C-16′′ (2x)).

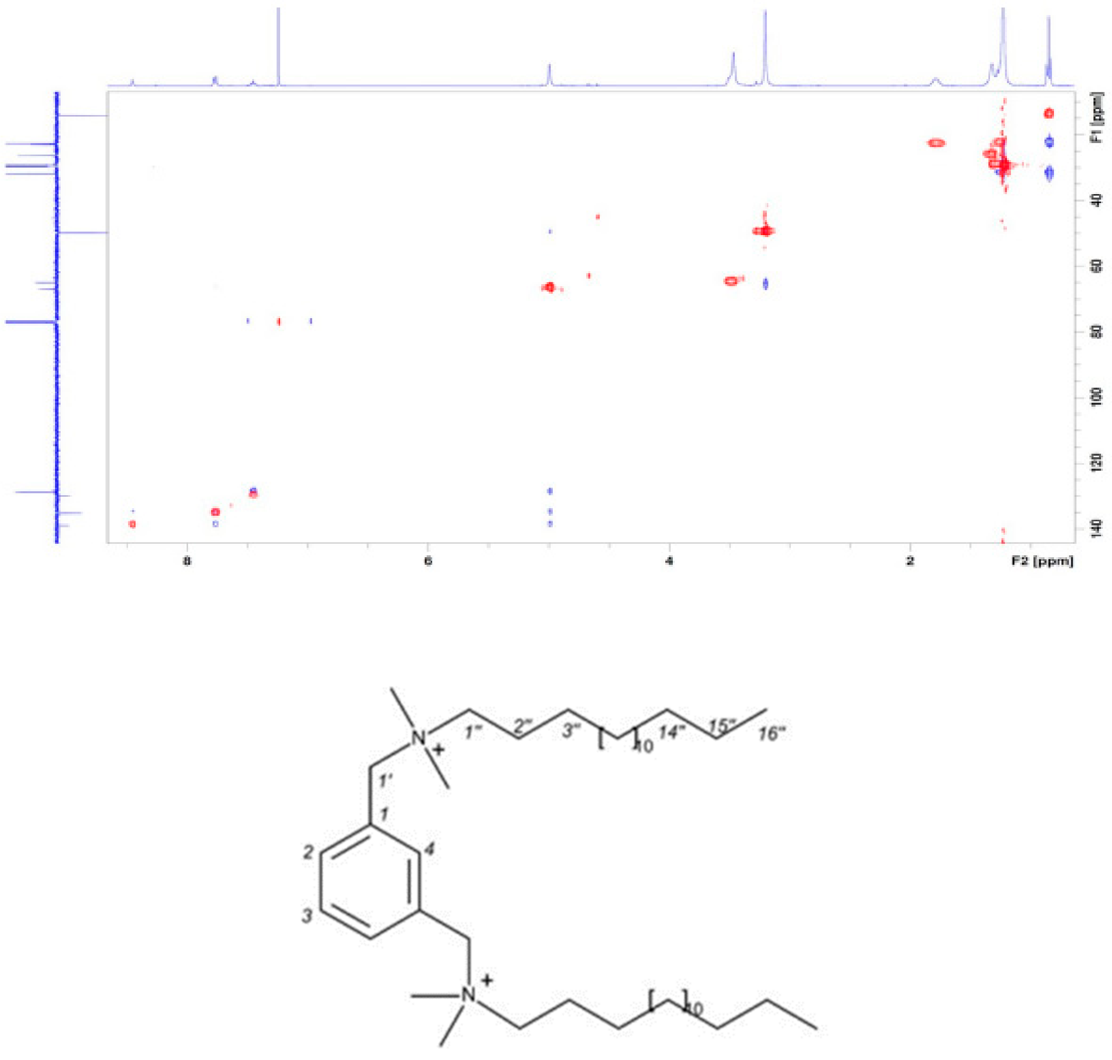

Figure S2. Combined HSQC (red) and HMBC (blue) spectra of compound m-16-Ph-16, together with the respective 1H and 13C NMR spectra on the axes and structure of m-16-Ph-16 compound.

Numbering is in accordance with that used for NMR shift data. It is not in accordance with IUPAC nomenclature but allows an easy comparison of different shifts in the compound.

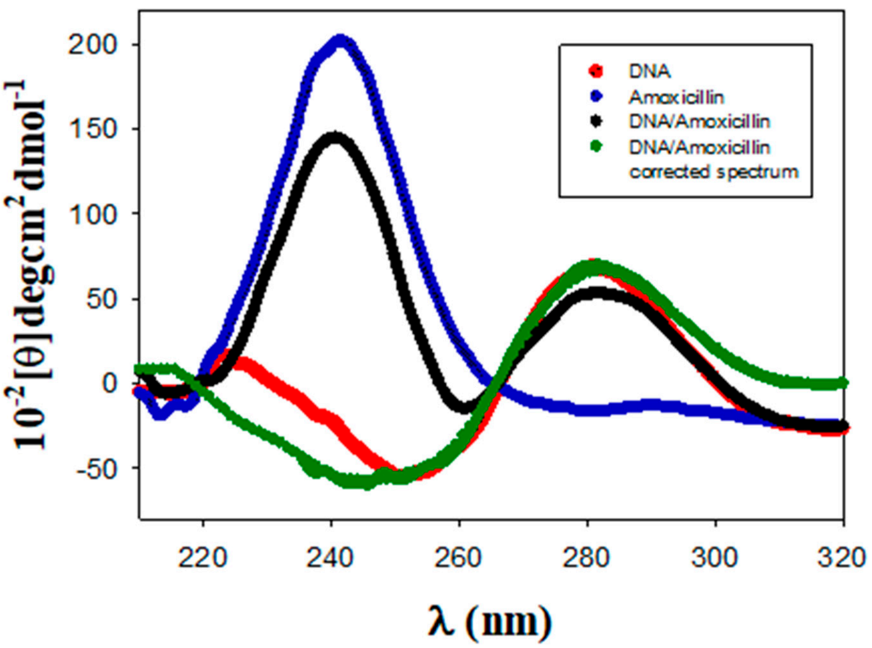

Figure S3. CD spectra in molar ellipticity units vs. wavelength in nm. (•) C_{DNA} = 68 μM, C_{AMOX} = 0 μM (•) C_{DNA} = 0 μM, C_{AMOX} = 68 μM, (•) C_{DNA} = 68 μM and C_{AMOX} = 68 μM, DNA/AMOX experimental spectrum (•) C_{DNA} = 68 μM and C_{AMOX} = 68 μM, DNA/AMOX corrected spectrum.

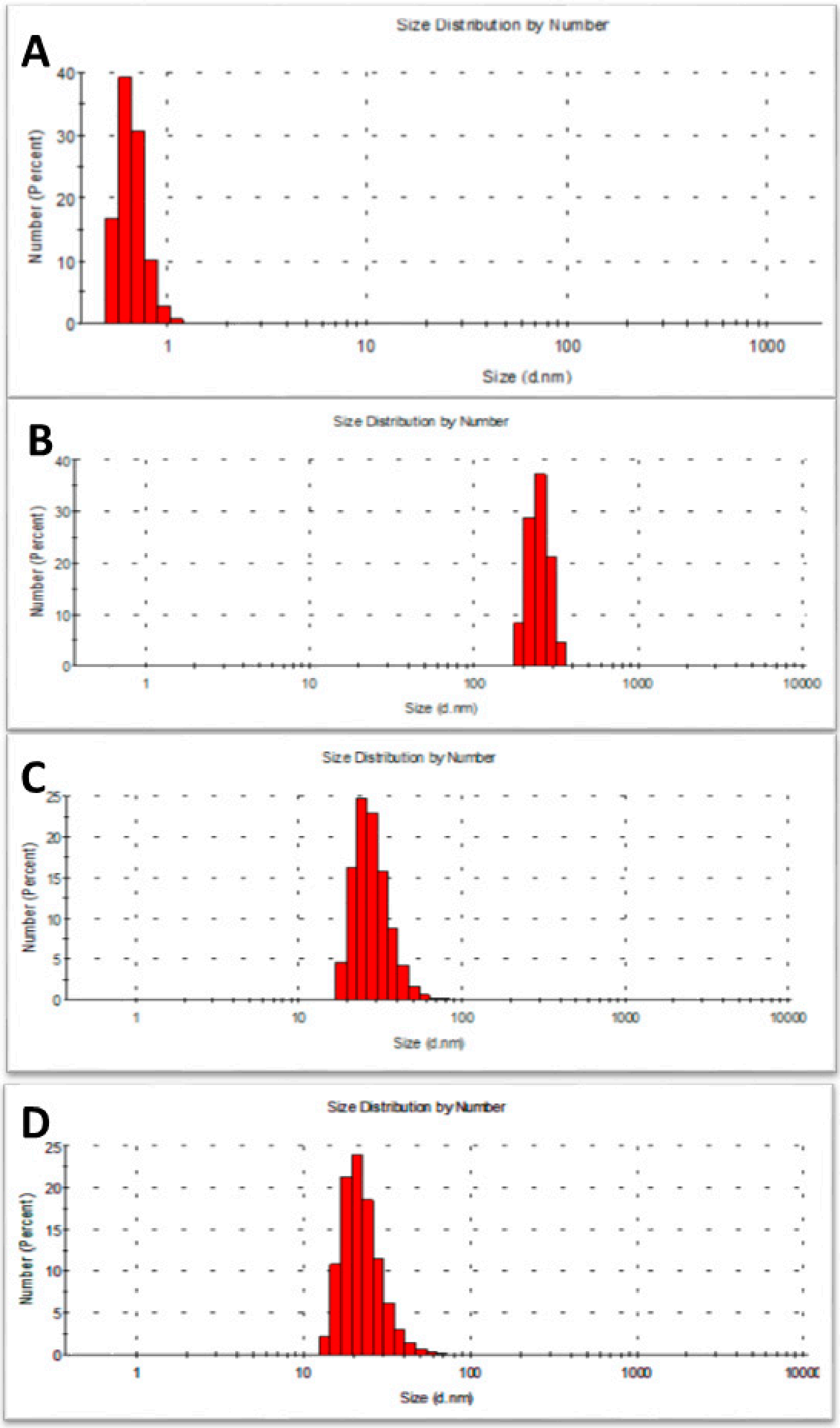

Figure S4. DLS size distribution by number of Au@16-mph-16, DNA/AMOX complex and Au@16-mph-16/DNA-AMOX nanosystems in water. (A) Au@16-mph-16, $C_{Au@16\text{-}mph\text{-}16}$ = 3.3 nM (B) DNA/AMOX complex, $C_{Au@16\text{-}mph\text{-}16}$ = 0 nM (C) NS_1, $C_{Au@16\text{-}mph\text{-}16}$ = 3.3 nM and (D) NS_2, $C_{Au@16\text{-}mph\text{-}16}$ = 33.3 nM. A fixed concentration of DNA and TC (C_{DNA} = 68 μM and C_{AMOX} = 68 μM) was used for the preparation of the nanocomplexes.

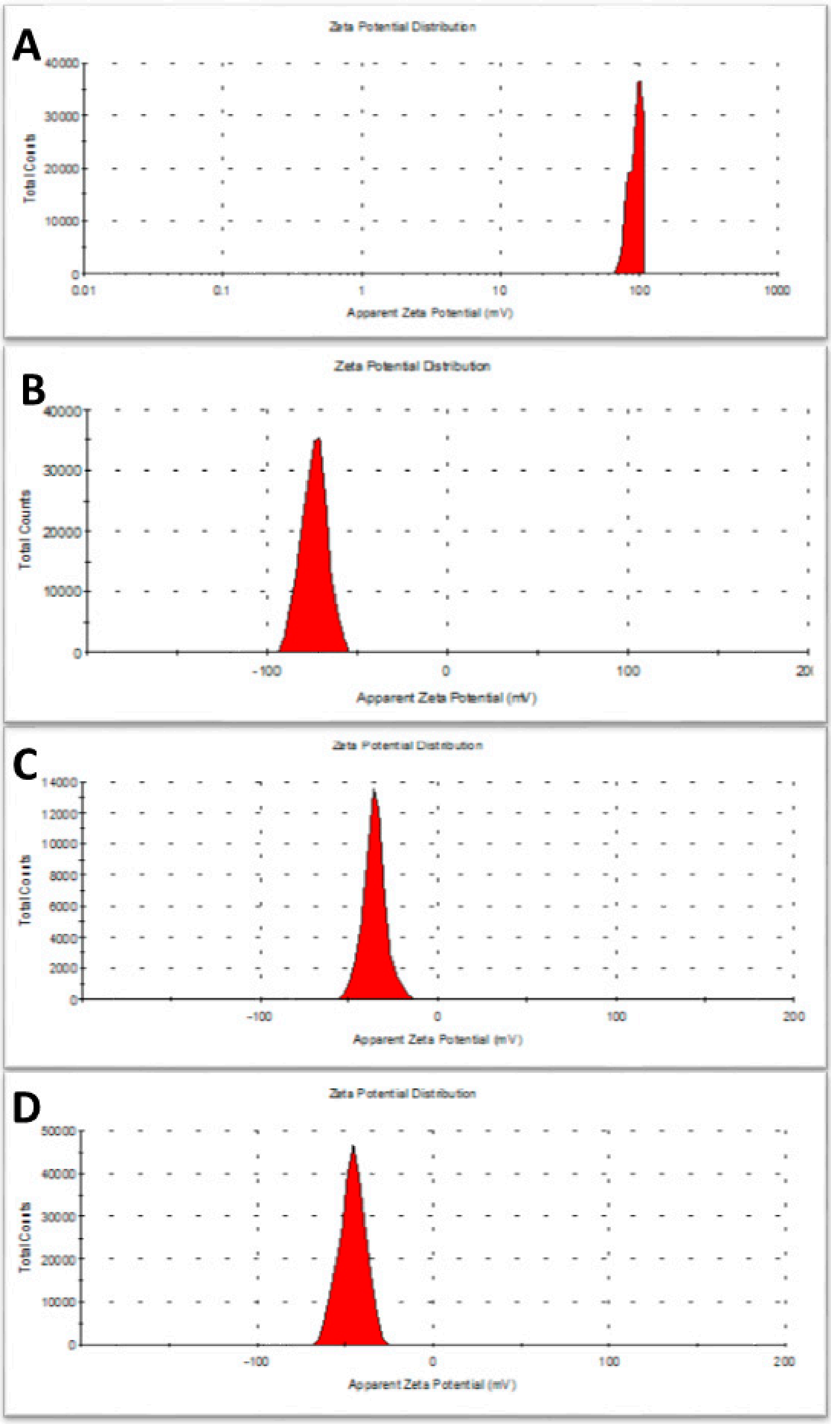

Figure S5. Zeta potential of Au@16-mph-16, DNA/AMOX complex and Au@16-mph-16/DNA-AMOX nanosystems in water. (A) Au@16-mph-16, $C_{Au@16\text{-}mph\text{-}16}$ = 3.3 nM (B) DNA/AMOX complex, $C_{Au@16\text{-}mph\text{-}16}$ = 0 nM (C) NS_1, $C_{Au@16\text{-}mph\text{-}16}$ = 3.3 nM and (D) NS_2, $C_{Au@16\text{-}mph\text{-}16}$ = 33 nM. A fixed concentration of DNA and AMOX (C_{DNA} = 68 µM and C_{AMOX} = 68 µM) was used for the preparation of the nanocomplexes.

.

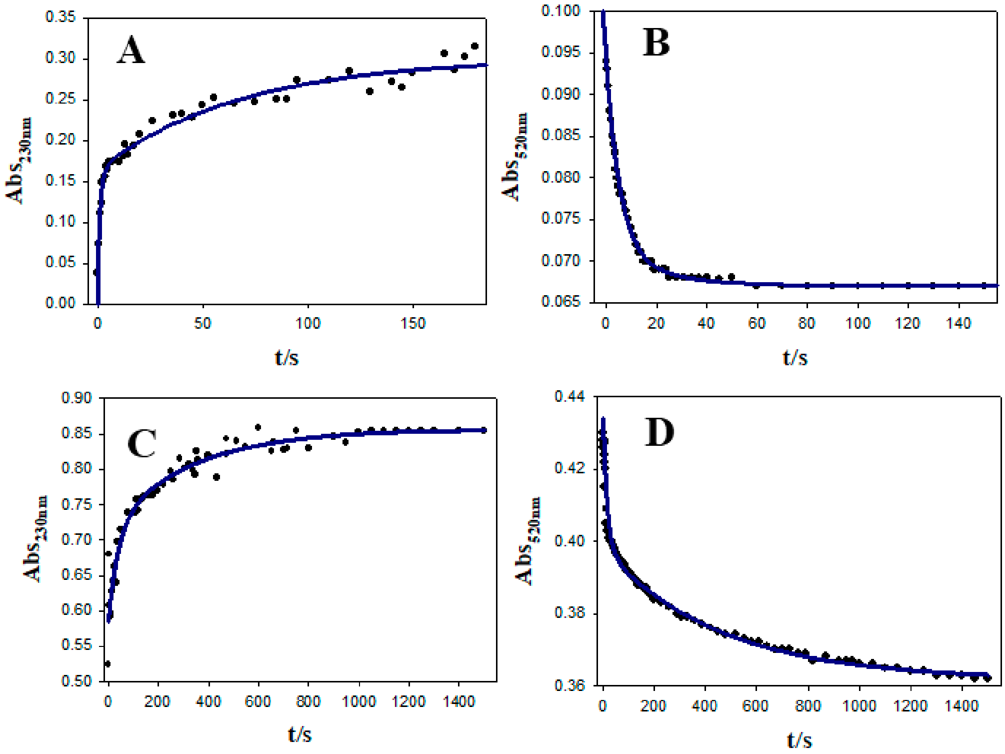

Figure S6. Examples of the kinetic curves for the release of AMOX in Müller-Hinton (MH) media at 37 °C from the nanosystems in two positions in UV-visible spectra: absorbance at 230 nm and 520 nm. The continuous line has been calculated according to a biexponential data fit. (A-B) NS_1 nanosystem and (C-D) NS_2 nanosystem.

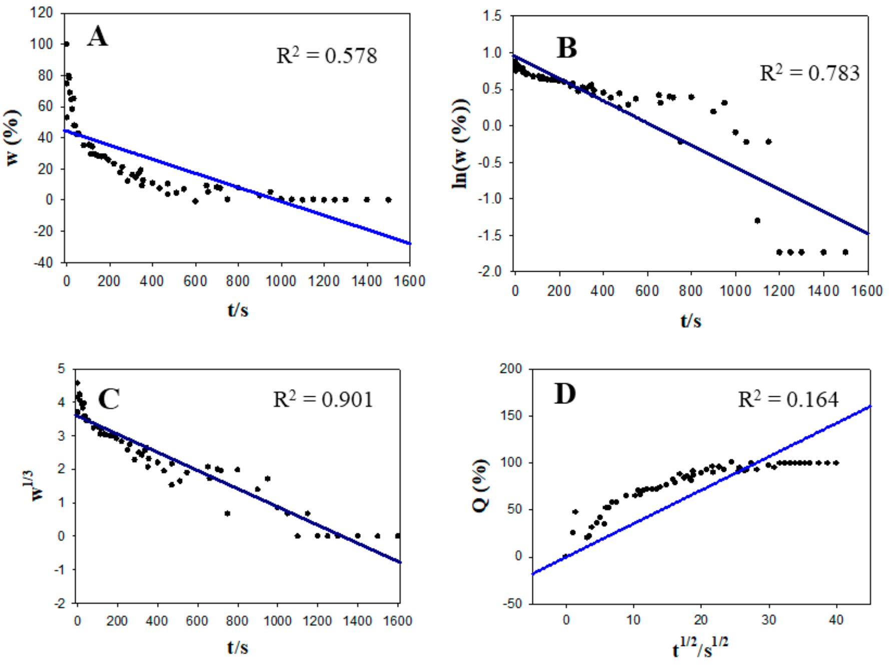

Figure S7. Release kinetics of AMOX from NS_2 formulation and the corresponding correlation coefficients. Continuous lines are the best fit of the kinetics data to distinct kinetic models. (A) Zero-order, (B) First-Order, (C) Hixson-Crowell, and (E) Higuchi model.

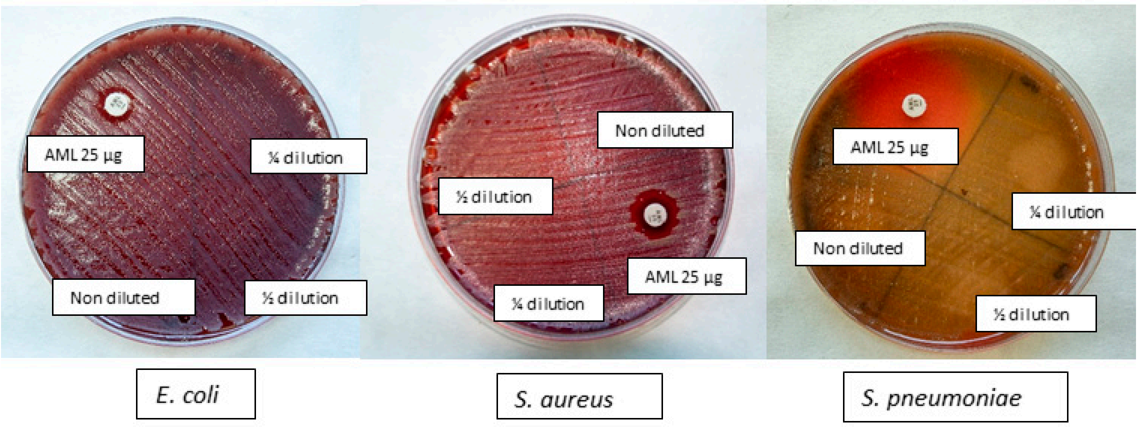

Figure S8. Effect of Amoxicillin disk (25 µg) and nanoparticle (non diluted and ½ - ¼ dilution) on bacterial cultures.

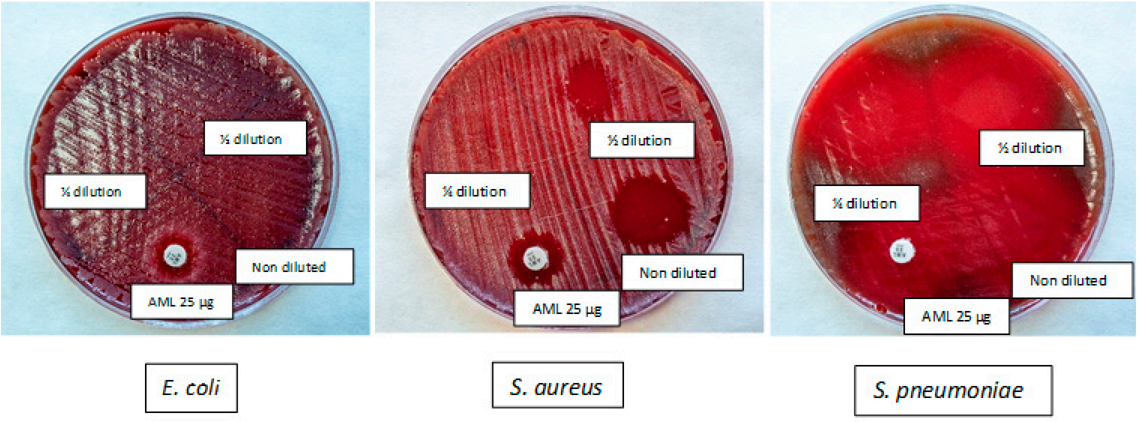

Figure S9. Effect of Amoxicillin disk (25 µg) and nanosystem (non diluted and ½ - ¼ dilution) on bacterial cultures.

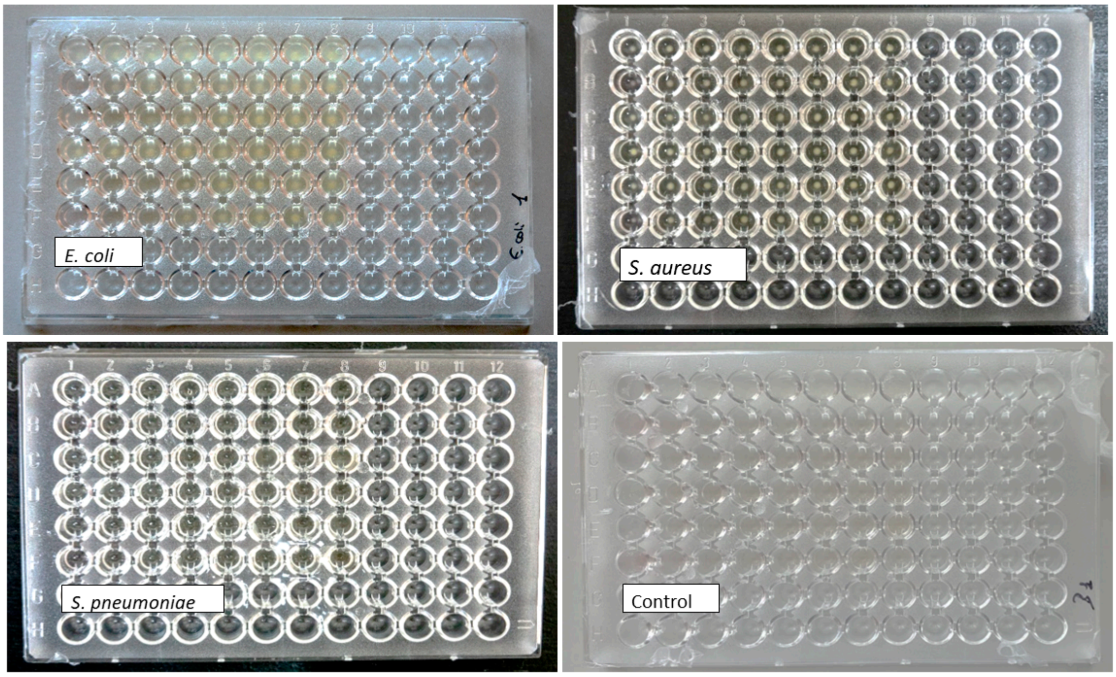

Figure S10. Microdilution plates assays, with MIC results for *E. coli*, *S. aureus*, *S. pneumonia* and control with Müller Hinton media.
